# Supplementary figures and images for: Extracting interpretable signatures of whole-brain dynamics through systematic comparison
Source: PLoS Comput Biol. 2024 Dec 23;20(12):e1012692. doi: 10.1371/journal.pcbi.1012692 (PMC11706466; doi:10.1371/journal.pcbi.1012692)

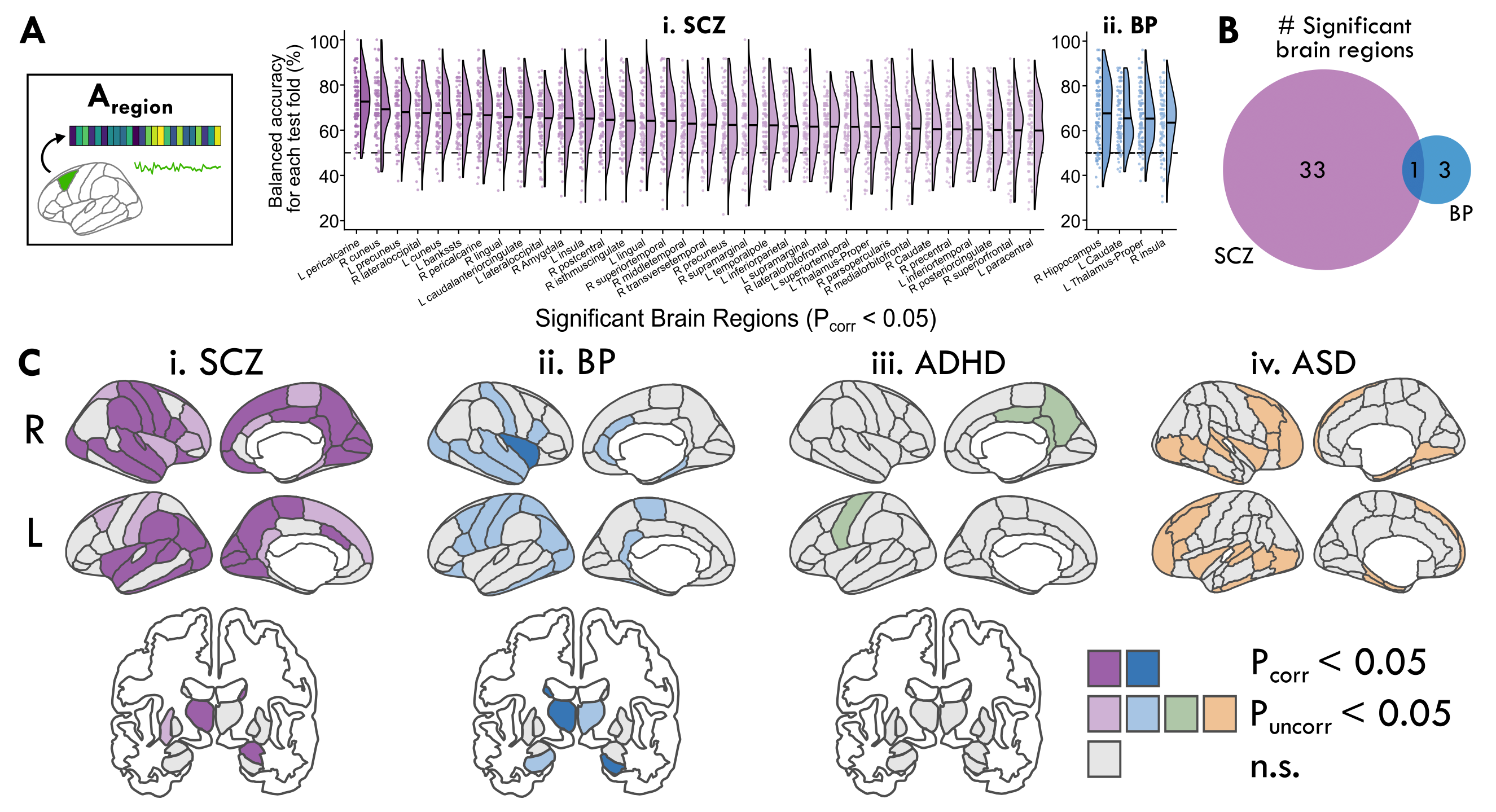

Supplement: S1 Fig — A. The distribution of balanced accuracy values across test folds is shown as a raincloud plot for each significant brain region (Padj < 0.05, corrected across 82 regions) in SCZ (i) and BP (ii). The horizontal line within each half-violin indicates the mean balanced accuracy for the corresponding brain region. B. The Venn diagram illustrates the number of significant brain regions for each of SCZ (purple) and BP (blue), indicating that one brain region (left thalamus) is shared between the two disorders. C. For each of the four disorders, regions are shaded dark to indicate Padj < 0.05 (corrected across 82 regions for SCZ, BP, and ADHD; across 48 regions for ASD). Additionally, regions are shaded light to indicate that the nominal uncorrected P < 0.05. Gray shading indicates that the uncorrected P > 0.05 for the balanced accuracy in the given region. (TIFF) [file pcbi.1012692.s001.tiff]

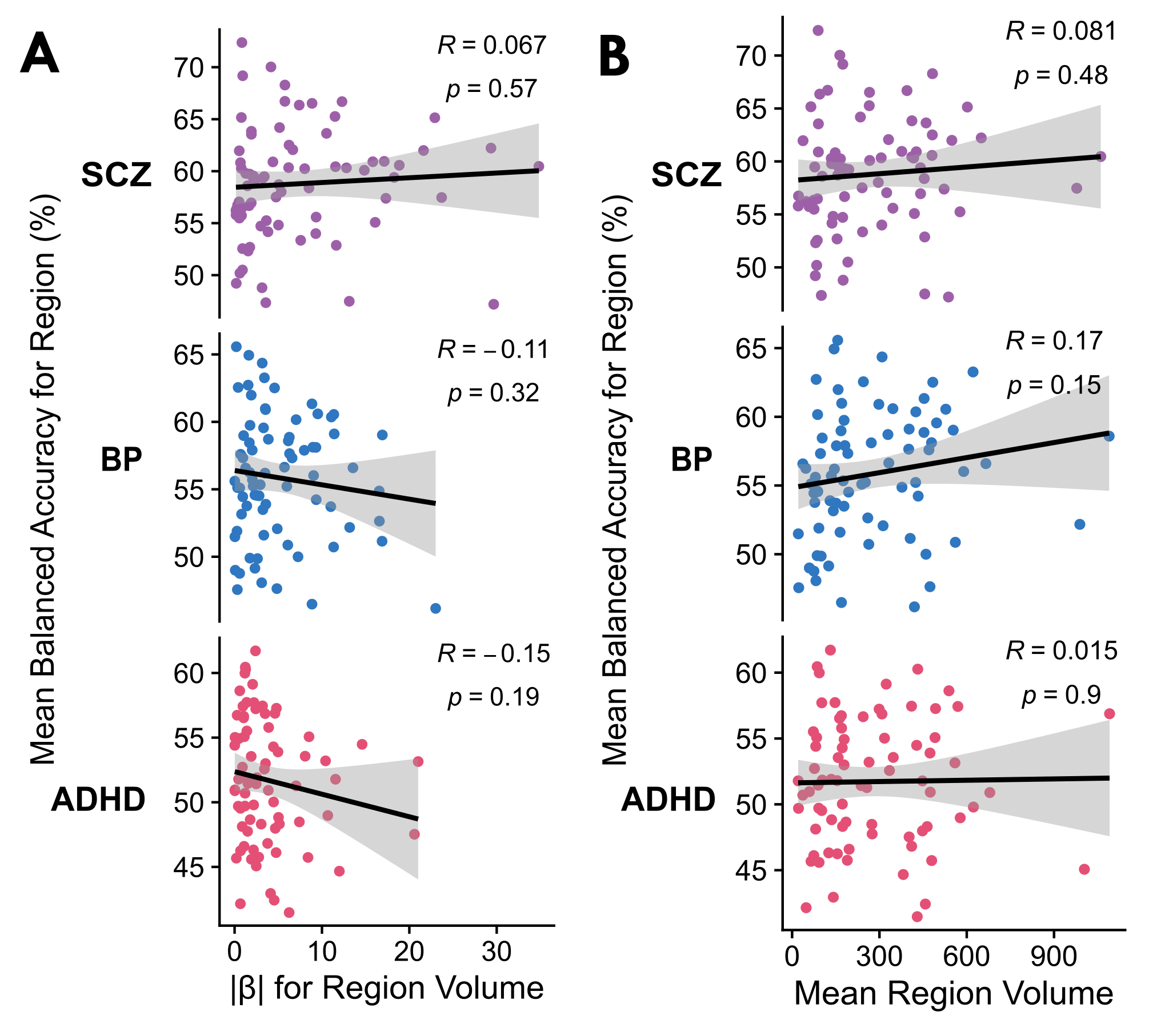

Supplement: S2 Fig — A. For each brain region in the UCLA CNP dataset, the mean balanced accuracy is plotted relative to the absolute β coefficient estimated from ordinary least squares regression of region volume on diagnosis per clinical group. Pearson correlation estimates (R) and corresponding P-values are annotated in the top right corners. B. As in A, for each brain region, the mean balanced accuracy is plotted relative to the average region volume (measured in number of voxels) across all participants in the UCLA CNP cohort. Pearson correlation estimates, R, and corresponding P-values are shown in the top right corners. (TIFF) [file pcbi.1012692.s002.tiff]

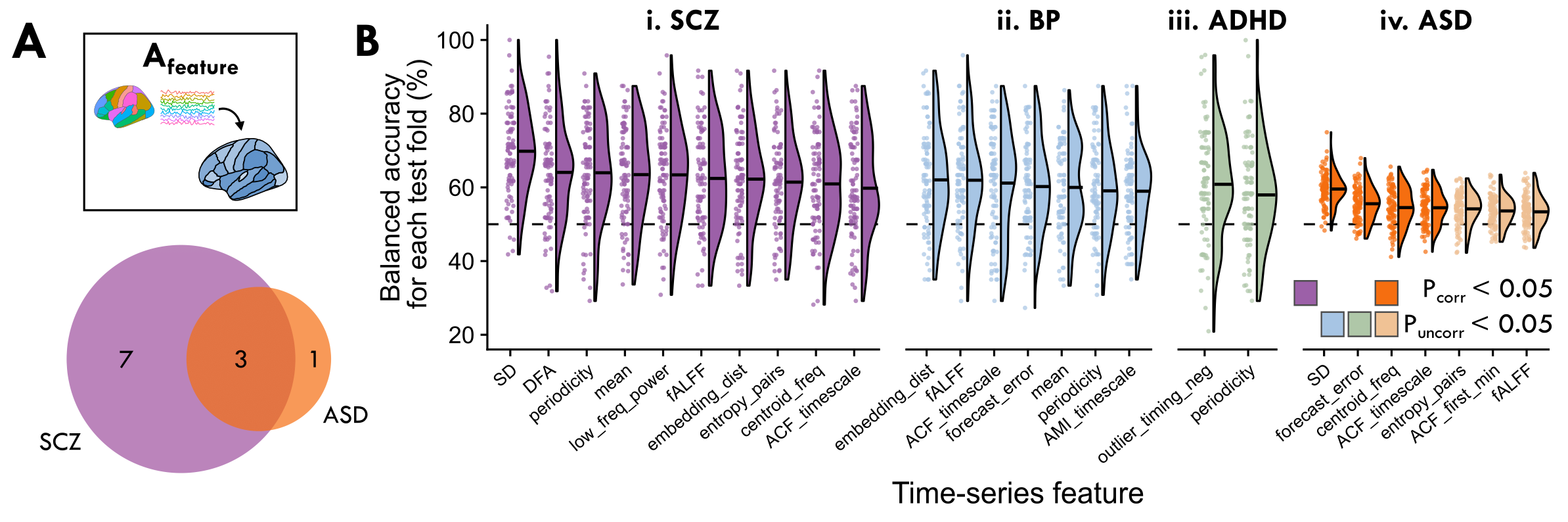

Supplement: S4 Fig — A. The Venn diagram illustrates the number of significant intra-regional time-series features for each of SCZ (purple) and ASD (orange), indicating that three features are shared between the two disorders. B. The distribution of balanced accuracy values across test folds is shown as a raincloud plot for each intra-regional time-series feature that yielded a balanced accuracy with either Pcorr < 0.05 (darker) or Puncorr < 0.05 (lighter). The horizontal line within each half-violin indicates the mean balanced accuracy for the intra-regional time-series feature. (TIFF) [file pcbi.1012692.s004.tiff]

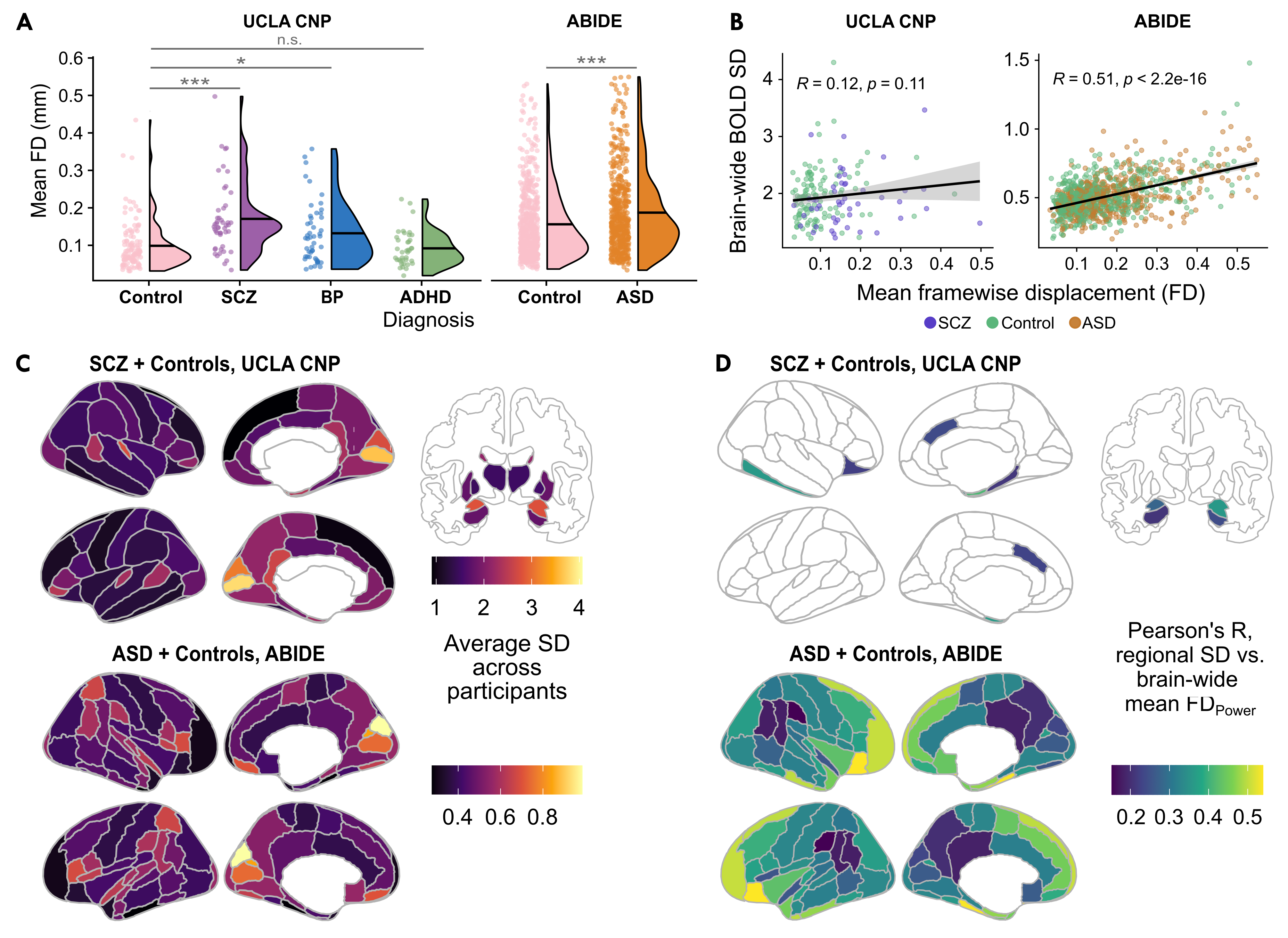

Supplement: S5 Fig — A. The mean framewise displacement (FD) computed with the method from [71] is shown with raincloud plots for all participants in the UCLA CNP (left) and ABIDE cohorts (right). The horizontal line within each half-violin indicates the mean FD for the corresponding group. Mean FD distributions were compared for each disorder relative to the corresponding control group using a Wilcoxon rank-sum test, with significance level indicated as *** P < 0.001, ** P < 0.01, * P < 0.05, n.s. P > 0.05. B. The brain-wide average BOLD SD is plotted against the mean FD across SCZ and control participants in the UCLA CNP cohort (upper) as well as ASD and control participants in the ABIDE cohort (lower), with the Pearson correlation estimates (R) and corresponding p-values shown in each plot. B. For each brain region, the average BOLD SD is shown across all SCZ and control participants in the UCLA CNP cohort (upper) as well as ASD and control participants in the ABIDE cohort (lower). Note that different color scales are used for the two cohorts, respectively. D. For each brain region, we computed the Pearson correlation between the region-wise BOLD SD and whole-brain mean FD values in the UCLA CNP cohort (upper) as well as ASD and control participants in the ABIDE cohort (lower). Pearson correlation estimates (R) are shown in brain maps, in which only brain regions for which Benjamini–Hochberg corrected P < 0.05 are shaded (corrected across 82 regions for UCLA CNP and 48 regions for ABIDE). Note that the same color scale is used for both cohorts. (TIFF) [file pcbi.1012692.s005.tiff]

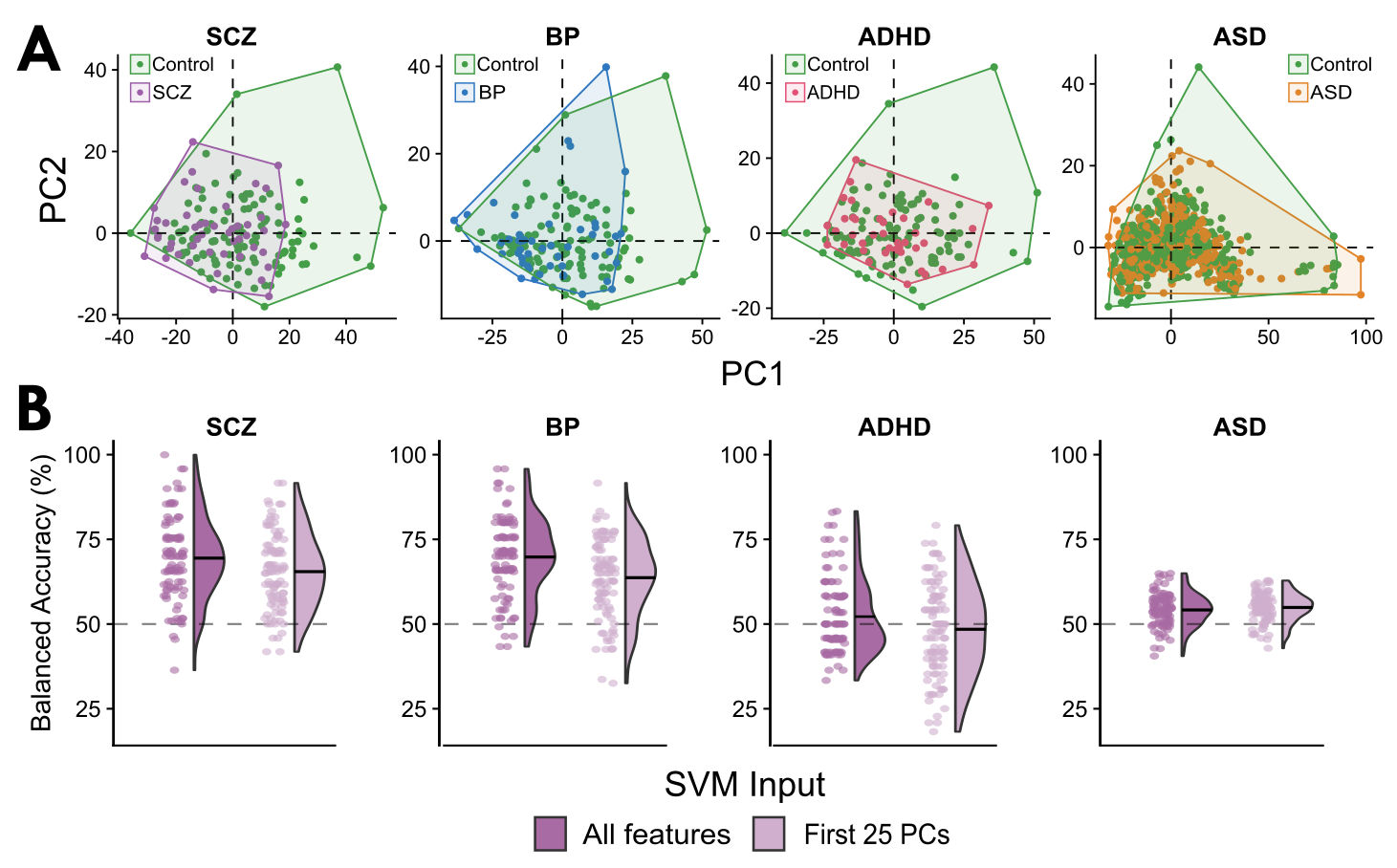

Supplement: S6 Fig — A. For each disorder, individual scores for the first two PCs are plotted, with points colored according to diagnosis. Shaded areas reflect convex hulls encapsulating all points for each diagnostic group. Note that each PCA was computed separately for each case–control comparison, so PC1 and PC2 scores are not directly comparable across clinical groups. B. For each case–control comparison, we compare the out-of-sample balanced accuracy across the 100 repeats × folds using all region × feature variables (left, dark purple) versus using only scores for the first 25 PCs (right, light purple). Points are randomly jittered along the horizontal axis in each raincloud plot to aid visualization. The horizontal line within each half-violin indicates the mean balanced accuracy for the corresponding distribution. C. For each case–control comparison, we compare the out-of-sample balanced accuracy across the 100 repeats×folds using default regularization (left, dark green) versus L1 (‘LASSO’ [90]) regularization (right, light green). (TIFF) [file pcbi.1012692.s006.tiff]

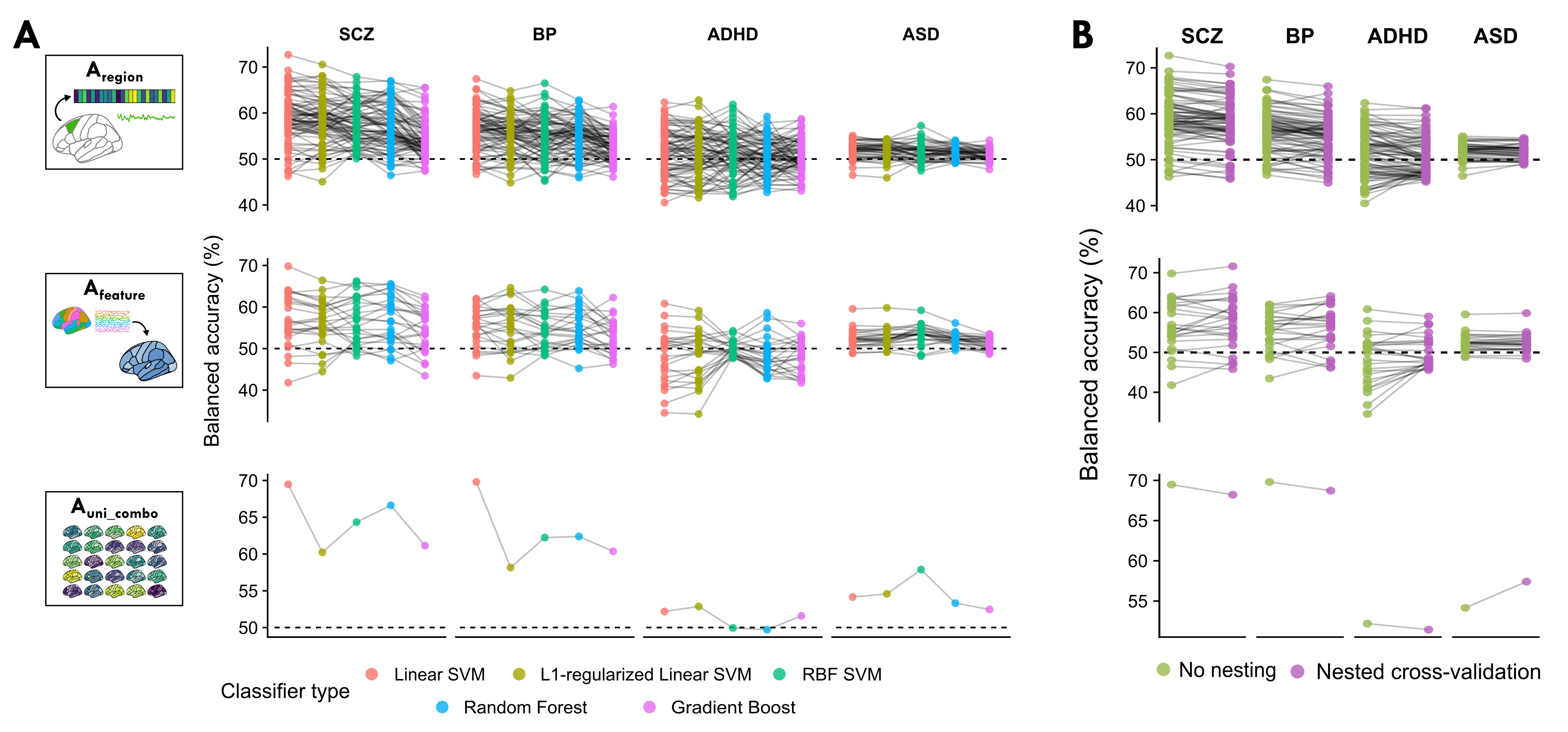

Supplement: S7 Fig — A. For each of the three univariate representations—Aregion, Afeature, and Auni_combo—the mean cross-validated balanced accuracy is shown per disorder using each of five different classifier types. Each dot corresponds to one model input type (e.g., left pericalcarine cortex in Aregion) and lines connect model input types across classifiers per disorder to guide visual interpretation. The dashed horizontal lines indicate 50% balanced accuracy in all plots. B. For the same univariate representations as in A, the mean cross-validated balanced accuracy is shown for the linear SVM classifier without hyperparameter optimization (i.e., explicitly setting C = 1 and applying inverse probability weighting; green) or with hyperparameter optimization for the C parameter and sample weighting type in purple. The dashed horizontal lines indicate 50% balanced accuracy in all plots. (TIFF) [file pcbi.1012692.s007.tiff]

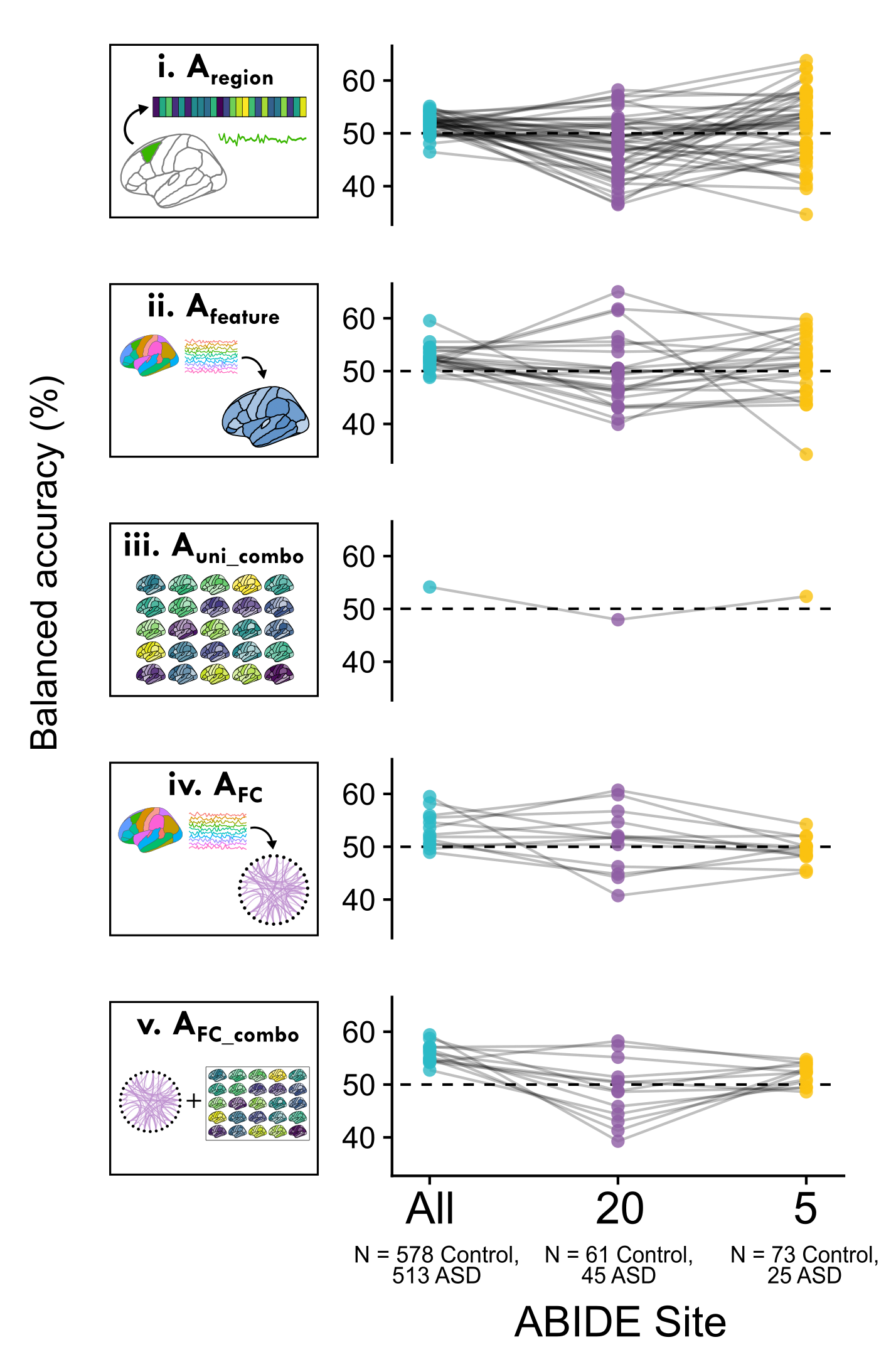

Supplement: S8 Fig — The mean cross-validated balanced accuracy is shown with the inclusion of participants from all ABIDE sites together (blue, N = 1091 participants) for each of (i) Aregion, (ii) Afeature, (iii) Auni_combo, (iv) AFC, and (v) AFC_combo. The mean cross-validated balanced accuracy is also shown when we restricted classification analyses to each of the two largest ABIDE imaging sites: Site #20 (purple, N = 106 participants) and Site #5 (yellow, N = 98 participants). Each dot corresponds to one individual model (e.g., the Superior Frontal Gyrus in Aregion) and lines connect models across ABIDE site analyses to guide visual interpretation. The dashed horizontal line marks 50% balanced accuracy in all plots. (TIFF) [file pcbi.1012692.s008.tiff]

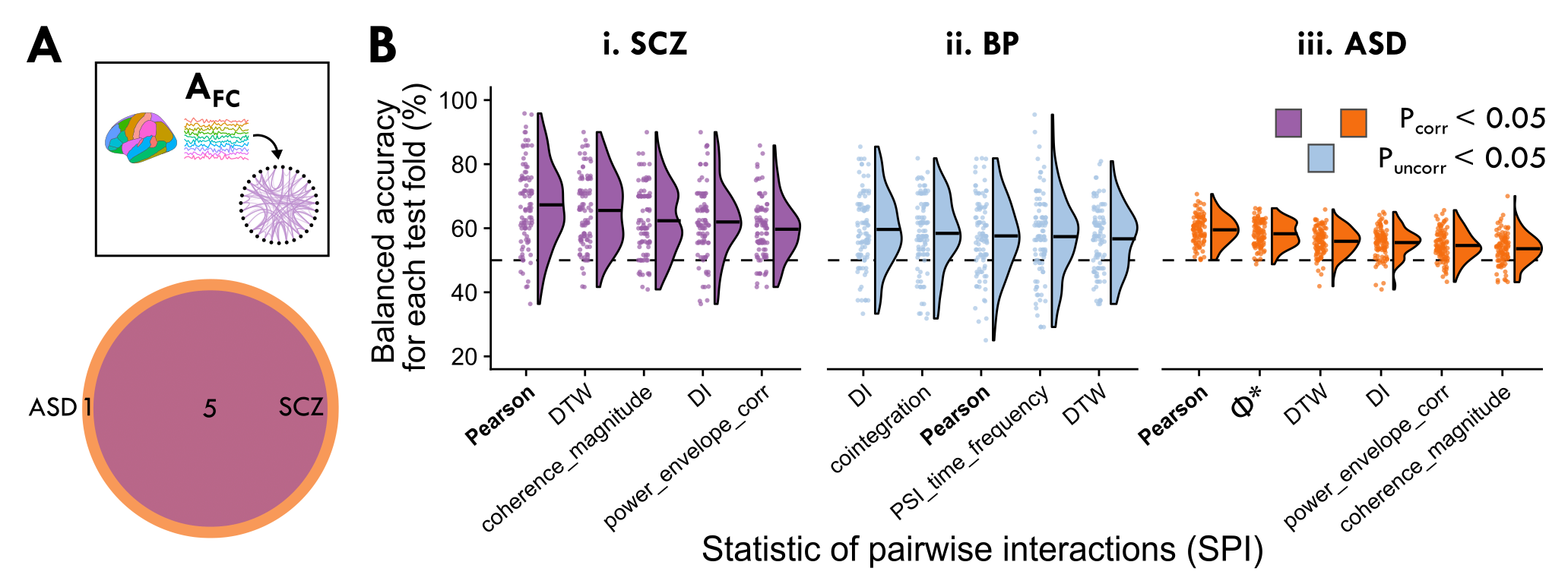

Supplement: S9 Fig — A. We compared 14 statistics of pairwise interactions (SPIs) (from pyspi [25], cf. Methods Sec. ‘Time-series feature extraction’), as different ways of quantifying functional connectivity (FC) between pairs of brain regions. For a given SPI, each participant was represented by the set of corresponding FC values (calculated for each pair of brain regions), yielding a set of region–region pair values that can be stored as a one-dimensional vector per participant. These vectors were concatenated to yield a participant × region–pair matrix that formed the basis for case–control classification using a linear SVM. B. Classification results are shown as a heatmap, with rows representing SPIs that yielded significant balanced accuracy (pcorr < 0.05, corrected across 14 SPIs) in at least one disorder, and columns representing each of the four disorders. Of the 14 SPIs we evaluated, eleven significantly distinguished cases from controls in at least one disorder, and are plotted here. The Pearson correlation coefficient is annotated in boldface for easier reference. C. The SPI similarity score, |ρSPI|, is visualized between each pair of the eleven SPIs from B as a heatmap, revealing six clusters of SPIs with similar behavior on the dataset (based on their outputs across all region–pairs and all disorders). As in B, the Pearson correlation coefficient annotation is shown boldface. D. The disorder similarity score, ρdisorder, is depicted to compare the balanced accuracy values among all 14 SPIs between each pair of neuropsychiatric disorders; a large positive ρdisorder indicates a strong positive Spearman correlation in case–control classification performance across the 14 SPIs in the given pair of disorders. (TIFF) [file pcbi.1012692.s009.tiff]

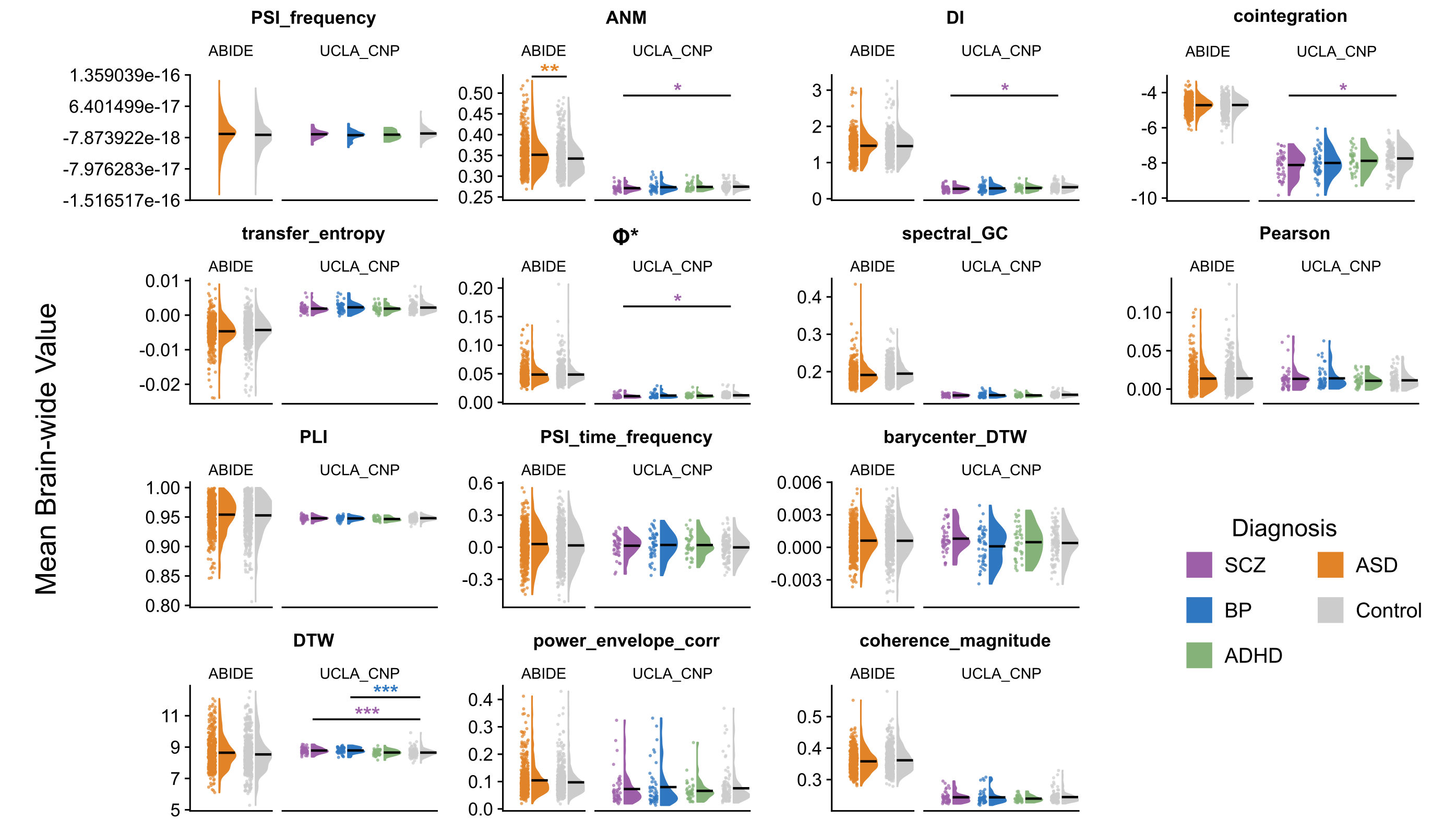

Supplement: S10 Fig — For each participant in the UCLA CNP and ABIDE cohorts, we calculated the mean FC value across all region–region pairs per SPI and show the distributions across participants as raincloud plots. The solid horizontal line in each half-violin indicates the mean balanced accuracy and the dashed horizontal axis line denotes 50% balanced accuracy. Wilcoxon rank-sum test results are indicated with ***, Pcorr < 0.001; **, Pcorr < 0.01; *, Pcorr < 0.05. (TIFF) [file pcbi.1012692.s010.tiff]

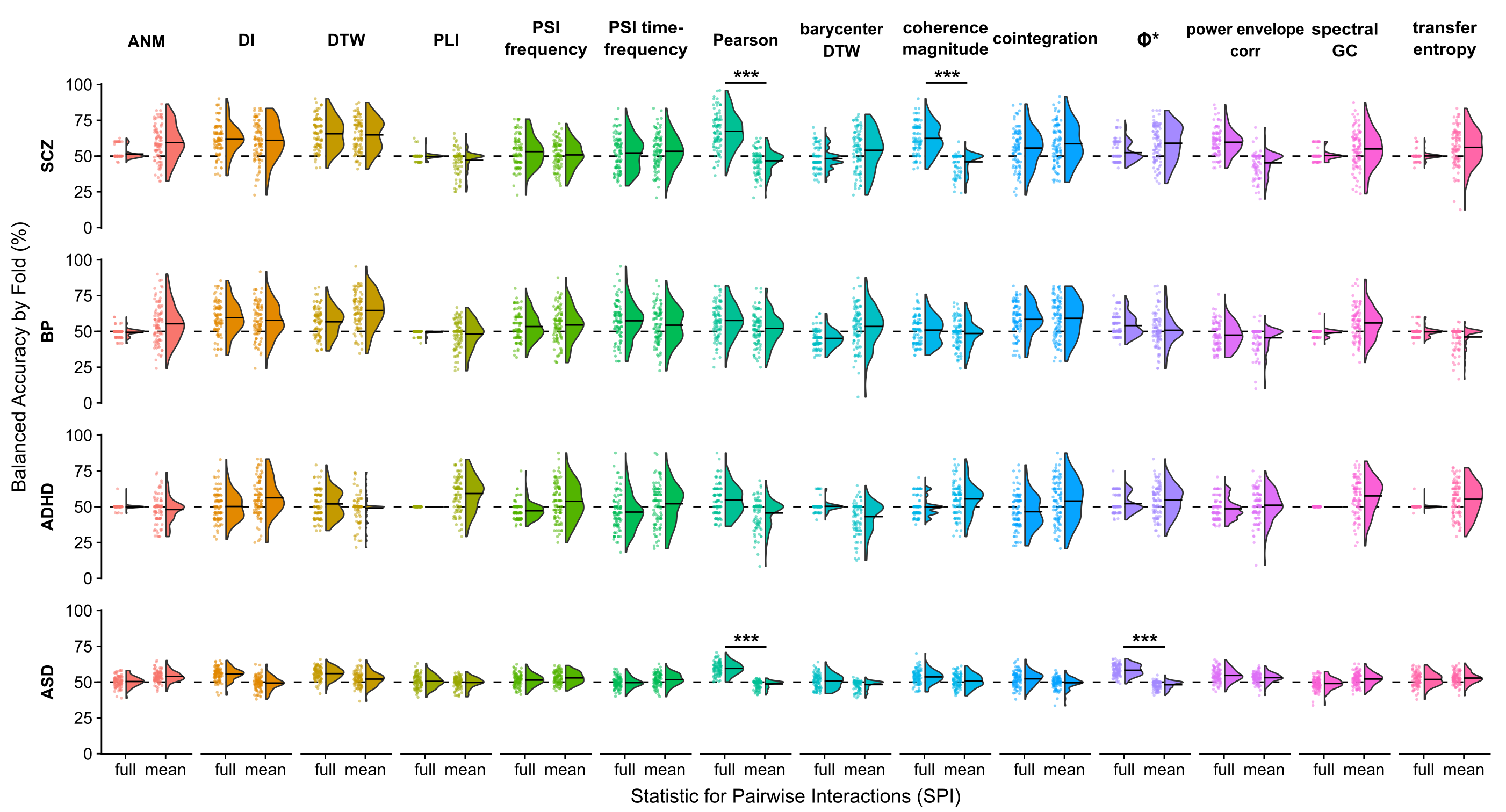

Supplement: S11 Fig — For each SPI, we compared case–control classification performance in each of the four disorders using either the full region–region pair input matrix (left) or the brain-wide average across all region–region pairs (right). Here, we show the distribution of all 100 test folds (10 repeats × 10 folds) as raincloud plots, where each dot represents one test fold. The solid horizontal line in each half-violin indicates the mean balanced accuracy and the dashed horizontal axis line denotes 50% balanced accuracy. Corrected resampled T-test results are indicated with ***, Pcorr < 0.001. (TIFF) [file pcbi.1012692.s011.tiff]

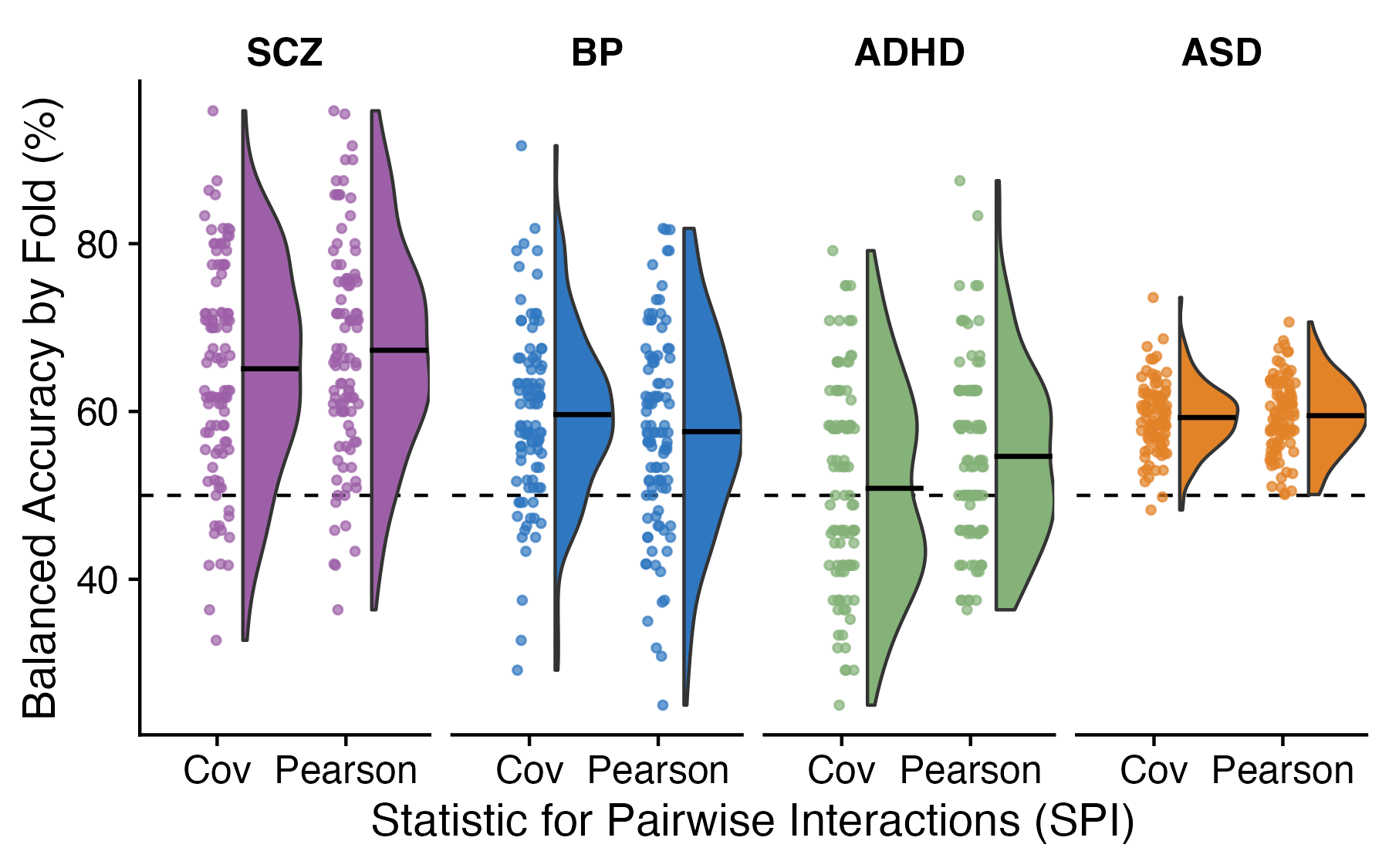

Supplement: S12 Fig — For each disorder (SCZ, BP, ADHD, and ASD), we plot the distribution of test balanced accuracy values across 100 folds (10 repeats × 10 folds) with the same scikit-learn pipeline for the raw covariance matrix across all region–pairs (‘Cov’, left) versus the normalized Pearson correlation matrix (‘Pearson’, right). The solid horizontal line in each half-violin indicates the mean balanced accuracy and the dashed horizontal axis line denotes 50% balanced accuracy. (TIFF) [file pcbi.1012692.s012.tiff]

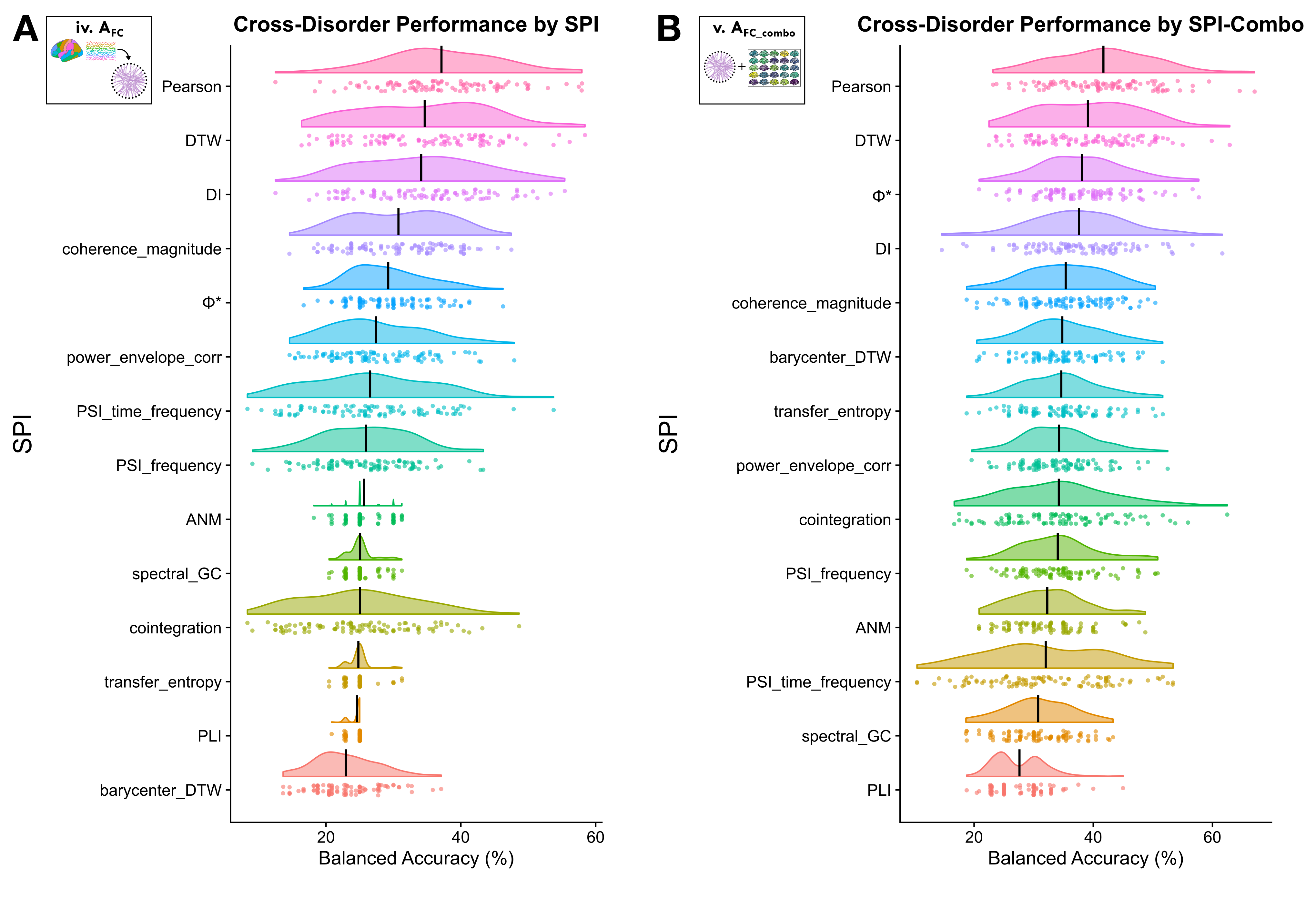

Supplement: S13 Fig — The out-of-sample balanced accuracy distribution (averaged across SCZ, BP, ADHD, and Control participants in the UCLA CNP cohort) is shown for each SPI on its own (A, AFC) or with the inclusion of whole-brain local dynamics (B, AFC_combo). Each dot corresponds to one test fold (for a total of 100 data points, across 10 repeats × 10 folds), and the vertical black line in each half-violin corresponds to the mean balanced accuracy across test folds. (TIFF) [file pcbi.1012692.s013.tiff]

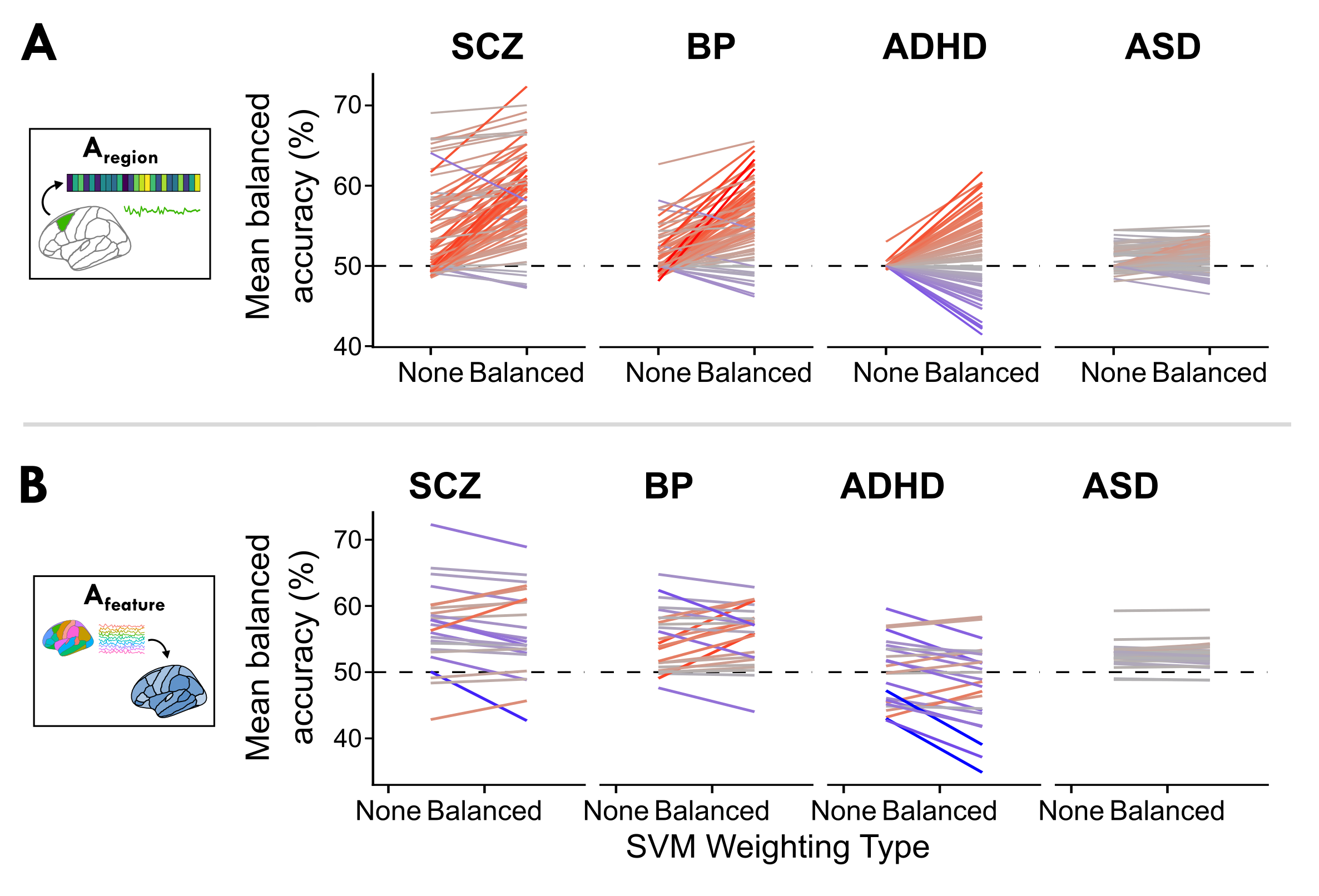

Supplement: S14 Fig — A. For each disorder, the mean balanced accuracy per brain region is shown with no weighting (‘None’) or inverse probability weighting (‘Balanced’). Lines correspond to each of 82 brain regions for SCZ, BP, and ADHD and each of 48 brain regions for ASD. Colors are included as a visual aid to highlight the difference in performance between the two weighting types, with red corresponding to higher balanced accuracy with inverse probability weighting and blue corresponding to lower balanced accuracy with inverse probability weighting. B. For each disorder, the mean balanced accuracy per univariate time-series feature is shown with no weighting (‘None’) or inverse probability weighting (‘Balanced’), as in A. (TIFF) [file pcbi.1012692.s014.tiff]

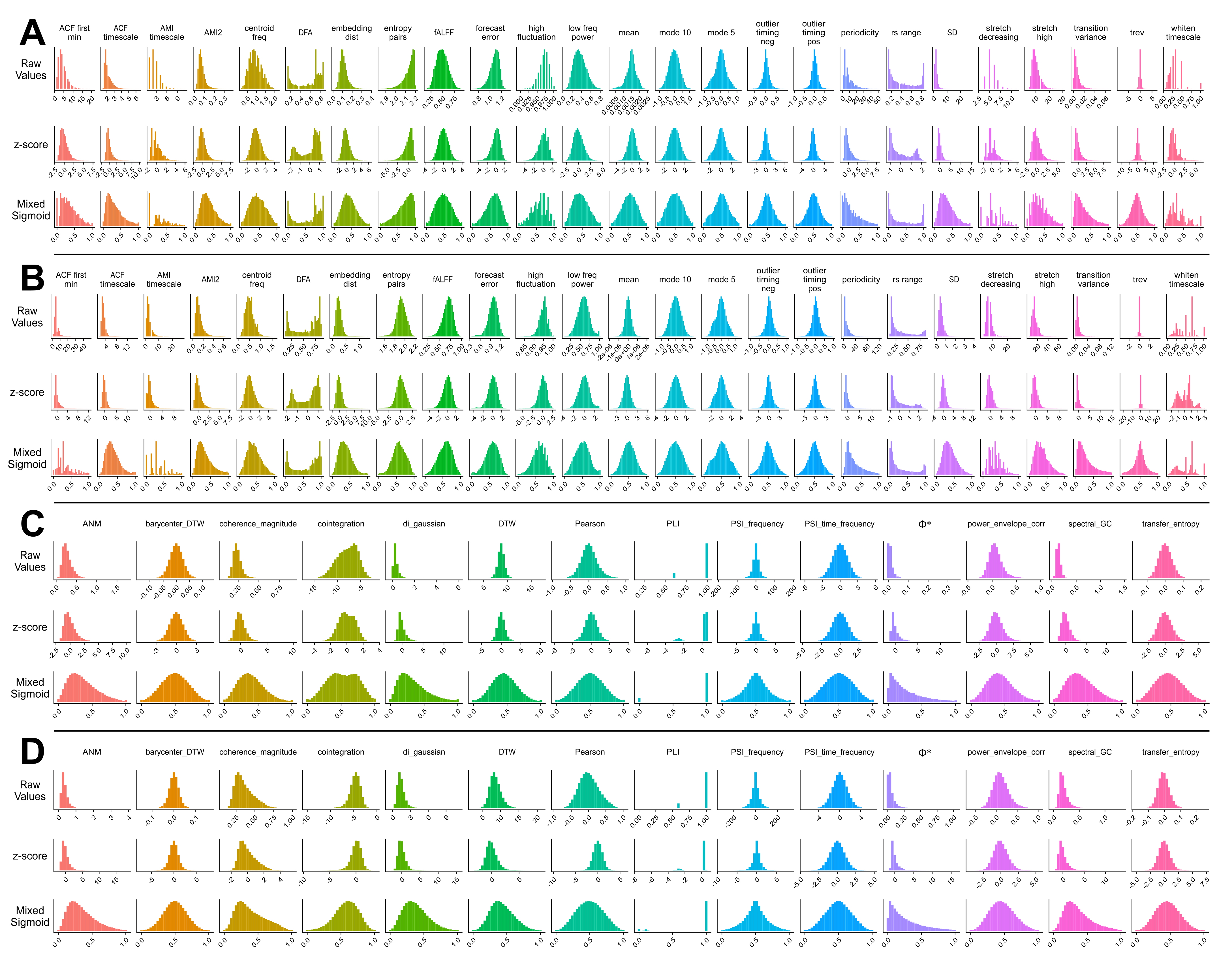

Supplement: S15 Fig — Univariate time-series feature values were concatenated from all brain regions, with the resulting distributions depicted for all participants in the UCLA CNP cohort (A) or ABIDE cohort (B) with no normalization (upper row), z-score normalization (middle row), and outlier-robust mixed sigmoid normalization (bottom row; see Methods Sec. ‘Case–control classification’ for description). Pairwise SPI feature values were concatenated from all region–region pairs, with the resulting distributions depicted for all participants in the UCLA CNP cohort (C) or ABIDE cohort (D) with no normalization (upper row), z-score normalization (middle row), and outlier-robust mixed sigmoid normalization (bottom row). (TIFF) [file pcbi.1012692.s015.tiff]

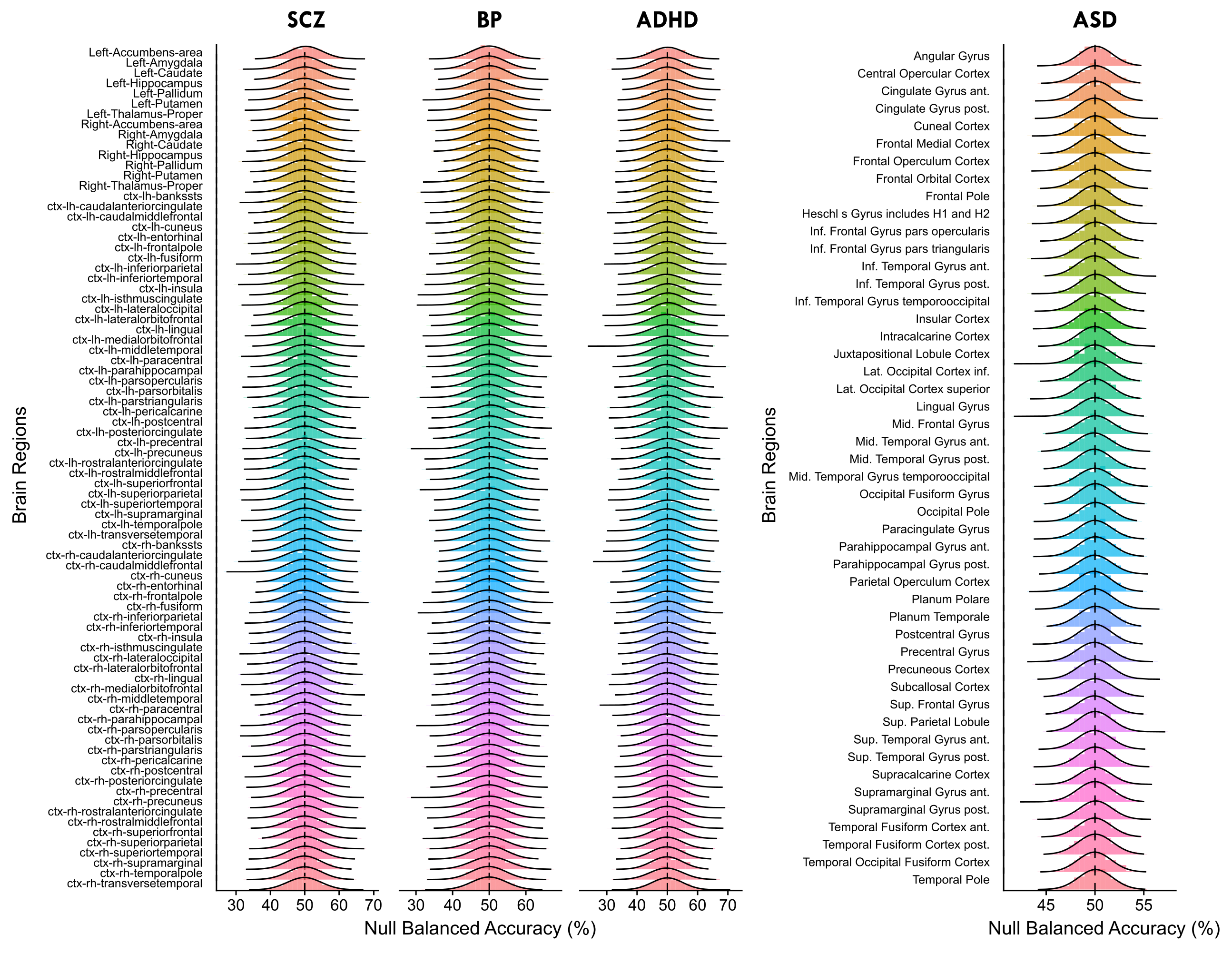

Supplement: S16 Fig — The distribution of 1000 null balanced accuracy values (cross-validated over 10 repeats of 10 folds each) is shown for each brain region by disorder. Empirical null values are visualized as histograms, with the probability density overlaid as black lines for the normal distribution based on the mean and standard deviation for each null distribution. These normal distributions were used to compute p-values for each brain region based on the corresponding probability density function; the same procedure was also applied for univariate and pairwise time-series features. ASD brain regions are plotted separately from the SCZ, BP, and ADHD brain regions as different parcellation atlases were analysed for UCLA CNP (Desikan-Killiany atlas) and ABIDE cohorts (Harvard-Oxford cortical altas). The dashed vertical line marks 50% balanced accuracy in all plots. (TIFF) [file pcbi.1012692.s016.tiff]
