# Supplementary material for: Extracting interpretable signatures of whole-brain dynamics through systematic comparison
Source: PLoS Comput Biol. 2024 Dec 23;20(12):e1012692. doi: 10.1371/journal.pcbi.1012692 (PMC11706466; doi:10.1371/journal.pcbi.1012692)
Supplement: Attachment — Submitted filename: Bryant_2024_PLOS_CB_R2R.pdf [file pcbi.1012692.s034.pdf]

Authors’ Response to Reviews of

Extracting interpretable signatures of whole-brain dynamics through systematic comparison

Annie G. Bryant, Kevin Aquino, Linden Parkes, Alex Fornito, Ben D. Fulcher
PLOS Computational Biology

R: Reviewer’s Comment, A: Authors’ Response, ☐ Manuscript Text

1. Reviewer #1

R: *In the present manuscript Bryant et al. applied intra- and inert-regional measures of resting-state (rs) fMRI data to make casecontrol classifications of four neuropsychiatric disorders. The authors showed that, while simple intra-regional statistics performed fairly well, combining intra-regional properties with inter-regional couplings generally improved the classification performance. I enjoyed reading the article, the work is clearly written, despite being very dense, and the methods are clearly presented. The results are interesting and consistent with previous findings and hypotheses. I think the proposed method will be very useful for other researchers studying large-scale brain activity, brain states, and neuropsychiatric disorders. Also, the method can be extended to include gene expression and neurotransmitter maps.*

A: We thank the reviewer for their thoughtful summary of the key findings of our manuscript and implications for expanding this method across domains.

R: *Nevertheless, I have some concerns that the authors should address to improve the manuscript:*

1.1. R1 comment #1

R: *It has been shown that "glocal" measures of rs-fMRI are useful for discriminating between brain states. Such measures combine local and global properties of brain activity. For instance, it has been shown that the coupling between each brain region and the rest of the cortex provides an efficient statistic for classifying wakefulness versus anesthesia-induced loss of consciousness (Ponce-Alvarez et al., 2022; Tanabe et al., 2020). The performance of this local-global measure should be studied in the present work and compared to the results obtained by combining intra- and inter-regional measures.*

A: We thank the reviewer for highlighting this particular aspect of brain functional organization—coupling between one brain region (local) and the rest of the brain (global)—and for providing relevant references for our exploration. We agree with the reviewer that statistical coupling between the activity of one brain region and that of the rest of the brain as described in Ponce-Alvarez et al. [1] represents an exciting avenue for research. To that end, we computed the Pearson correlation R between the BOLD signal for each brain region and the averaged time series from the rest of the brain (including subcortical regions for SCZ, BP, and ADHD), forming a ‘local–global’ coupling statistic for each brain region. This statistic generally exhibited a Gaussian distribution across brain regions and participants within each diagnostic group, as depicted below in Fig. R1.

We evaluated the case–control classification performance for each brain region’s local–global coupling statistics in each of the four disorders (SCZ, BP, ADHD, and ASD) using the same 10-repeated 10-fold cross-validated linear support vector machine (SVM) framework as in our main analyses. The results of this are depicted below, in Fig. R2, finding that our set of 25 properties of local intra-regional dynamics generally

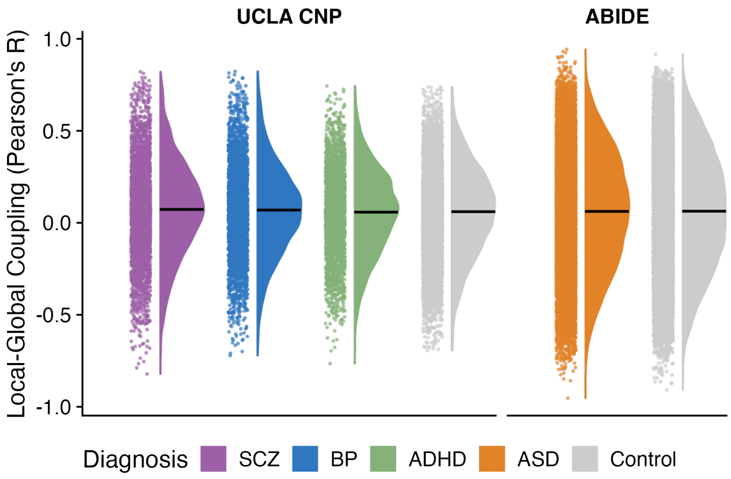

Figure R1: **Local–global coupling values across diagnostic groups.** For each diagnostic group in the UCLA CNP cohort (left) and ABIDE cohort (right), the distribution of local–global coupling statistics is shown across all brain regions and all participants. Each dot represents one brain region from one participant, and the horizontal line in each half-violin indicates the mean coupling statistic value (i.e., Pearson's R).

yield higher case–control classification performance. Note that our application of global signal regression (based on our empirical findings described previously [2]) precluded us from comparing voxelwise synchrony to the global signal, as in Tanabe et al. [3].

Our manuscript introduces a systematic method for comparing, as comprehensively as possible, the statistical properties of single time series (univariate measures) and pairs of time series (bivariate measures). We assess and interpret a total of 510 individual models across all examined brain regions, univariate time-series features, pairwise time-series features, and combinatorial models in the four disorders. As the reviewer notes, the result is a very analysis-"dense" manuscript, and we believe that it would complicate an already long and information-rich manuscript to extend its scope to incorporate major extensions into new classes of dynamical structure (such as local–global coupling). However, we agree that these are important and interesting directions that warrant further study, which we have added to an expanded Discussion section on fruitful directions in which to extend our work:

> Consistent with complex and spatially distributed disruptions to rs-fMRI activity, we found that all 14 SPIs better distinguished cases from controls for SCZ, BP, and ASD when we also included brain-wide maps of all 25 intra-regional time-series features in A_{FC_combo} (see Sec. 3.5). This finding is in line with previous work demonstrating that combining local and pairwise properties of rs-fMRI data can synergistically improve classification performance [4–7], supporting the notion that intra-regional activity and inter-regional coupling provide complementary information about disorder-relevant brain dynamics. While we cover an extensive space of dynamical statistics here, future applications might combine local dynamics with inter-regional coupling using network properties, local–global coupling, geometric embedding techniques like regional homogeneity (ReHo), or higher-order interactions. **For**

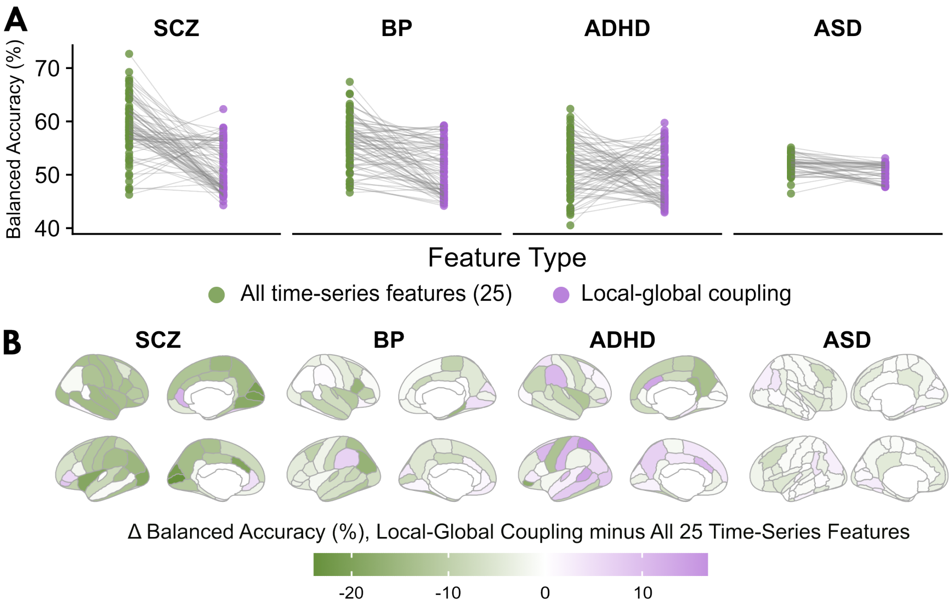

Figure R2: **Combining multiple properties of local intra-regional dynamics generally yields higher case–control classification performance.** **A** For each brain region per disorder, we compared the mean cross-validated classification performance with all 25 properties of intra-regional local dynamics (green) versus the single local–global coupling statistic (purple). Lines correspond to individual brain regions for comparison between the two feature types. **B** We computed the difference in balanced accuracy using local–global coupling versus all 25 properties of intra-regional local dynamics, with the resulting differences plotted in spatial brain maps.

example, Ponce-Alvarez et al. [1] and Tanabe et al. [3] demonstrated the benefits of quantifying 'glocal' measures [8] of synchrony between individual regions and whole-brain networks for distinguishing wakefulness from anesthetic states.

1.2. R1 comment #2

R: *In the same vein, the performance of global measures should be tested and discussed. For example: a) the average correlation between all pairs of regions and b) the metastability measure (i.e., the standard deviation of the order parameter of signal phases) (Cabral et al., 2011). These global measures have been widely used to characterize different brain states.*

A: We thank the reviewer for highlighting the use of global statistics of brain dynamics, including statistics of brain synchrony. As we describe above (R1.1), since there are myriad dynamical statistics that could be measured from a multivariate time series (ranging from local dynamics and pairwise coupling to distributed, global, and higher-order properties), our aim here was to be as comprehensive as possible in capturing the statistical properties within the constraint of looking at different types of (and combinations of) univariate and bivariate dynamical structure in multivariate time series. Nevertheless, we first note that some global properties that are derivable from the pairwise correlation structure are incorporated in our A_{FC} representation as special cases. A linear classifier applied to such a representation classifies a new time series based on

a trained linear combination of all SPI values; a special case is when all such values are equally weighted, yielding a value proportional to the mean FC. We now explicitly note that the A_{FC} representation allows the classifier to flexibly derive global properties from linear combinations of region pairs in our revised Results (relevant sentence in bold):

> We quantified pairwise communication as time-series dependencies, venturing beyond the traditional linear, contemporaneous Pearson correlation-based functional connectivity to compare its ability to distinguish cases from controls relative to thirteen other statistics of pairwise interactions (SPIs) from the *pyspi* library [9]. In total, these fourteen SPIs collectively measure different types of inter-regional coupling, including nonlinear, time-lagged, and frequency-based interactions, derived from literature including causal inference, information theory, and spectral methods (see Methods, Sec. 5.2.2 and Table 3 for more details). By comparing multiple different types of functional coupling, we aimed to investigate whether the Pearson correlation coefficient is comparatively a top performer, or if alternative metrics would be more sensitive to case–control differences in a given disorder. As depicted schematically in Fig. 1D(iv), each SPI was computed between the rs-fMRI time series of all region–region pairs per participant, and we concatenated values across all region–region pairs as classifier inputs for each SPI (see Methods, Sec. 5.3.1). **Supplying the full region–region pair space to the linear SVM allows the classifier to learn global properties of brain connectivity through linear combinations of all region pairs, including the brain-wide mean pairwise correlation as a special case [10]**.

The linear SVM classifier generalizes the mean-FC to flexibly identify the decision boundary among input features that maximizes the margin between the two given classes. We take the reviewer's point that the mean brain-wide FC value across SPIs is of interest, which we have visualized as a supplementary figure (Fig. S10) and reproduced below (cf. Fig. R3) for each SPI across participants in both the UCLA CNP and ABIDE cohorts.

As shown in Fig. S10 (Fig. R3 in this document), we identified several SPIs for which the mean brain-wide values are significantly different in a given case–control comparison (based on a Wilcoxon rank-sum test, applying Benjamini–Hochberg correction across the 14 SPIs per diagnostic group). For example, dynamic time warping (DTW) showed significantly higher global values in SCZ and BP than in control participants from the UCLA CNP cohort (both $P_{\mathrm{corr}} < 0.001$). Given these differences, we tested the classification performance of global brain-wide FC values for each SPI with the same 10-repeat 10-fold cross-validated linear SVM pipeline as with our main analyses. We now depict the fold-wise results in the supplements (Fig. S11), reproduced here as Fig. R4 below. We applied the same corrected two-tailed test statistic T_{corr} for repeated k-fold cross-validation as used in the main text (using the *correctR* package [11]) to compare performance distributions.

As shown in Fig. S11 (Fig. R4 in this document), we found only four instances of a statistically significant difference ($P_{\mathrm{corr}} < 0.05$) between the full input feature space versus the brain-wide mean: Pearson-based FC in SCZ and ASD, coherence magnitude-based FC in SCZ, and Φ^*-based FC in ASD. All four instances corresponded to significantly higher classification performance using the full region–region pair matrix that allowed the linear SVM to generalize beyond the mean of each set of input features. This supports our chosen focus on the more general setting in which the full region–region pair matrix is supplied to linear SVM classifiers in the manuscript. We now address this finding and its interpretation in the Results section of the revised manuscript (relevant sentence in bold):

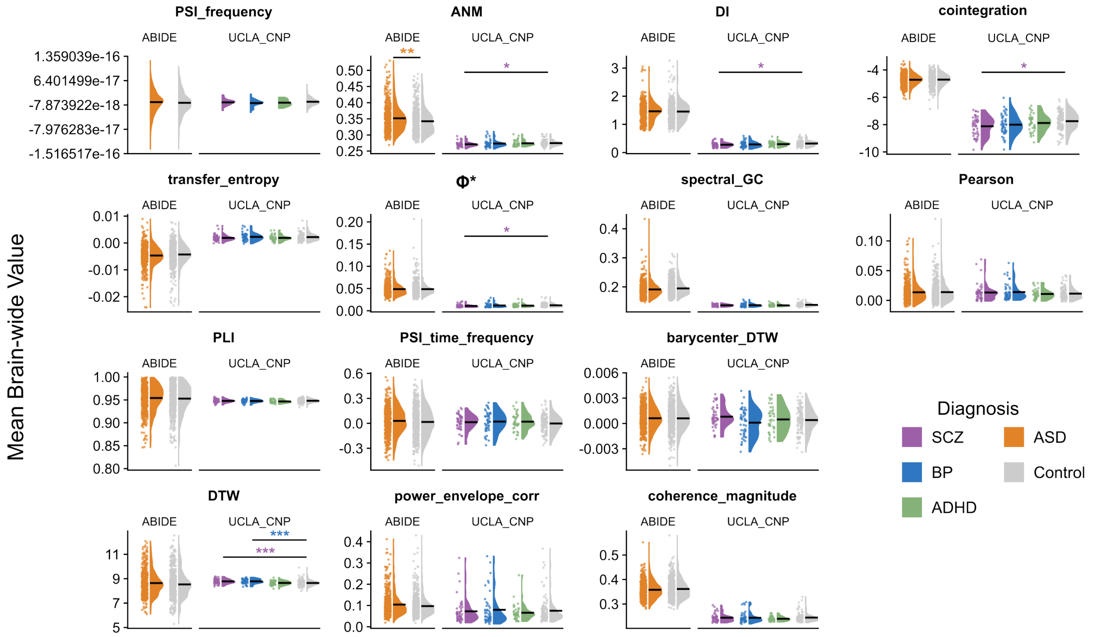

Figure R3: **The mean FC value across all region–region pairs by SPI.** For each participant in the UCLA CNP and ABIDE cohorts, we calculated the mean FC value across all region–region pairs per SPI and show the distributions across participants as raincloud plots. The solid horizontal line in each half-violin indicates the mean balanced accuracy and the dashed horizontal axis line denotes 50% balanced accuracy. Wilcoxon rank-sum test results are indicated with ***, $P_{\text{corr}} < 0.001$; **, $P_{\text{corr}} < 0.01$; *, $P_{\text{corr}} < 0.05$.

We found that for SCZ and ASD, representing rs-fMRI dynamics through pairwise coupling significantly distinguished cases from controls—for five SPIs in SCZ ($P_{\text{corr}} < 0.05$, one-tailed permutation test, corrected for 14 comparisons using the method of Benjamini–Hochberg) and six SPIs in ASD. The test fold–wise balanced accuracy distribution is shown for each SPI in SCZ and ASD in Fig. 3A (see Table S12 for all balanced accuracy results). By contrast, none of the fourteen SPIs significantly classified BP or ADHD cases from controls; the distributions for the top five performing SPIs in BP are visualized in Fig. S9B, although we focus on SCZ and ASD in this section. **Note that while some SPIs showed case–control differences in brain-wide average FC values (cf. Fig. S10), the global mean FC values performed similar or worse to classifiers supplied with the full region–region pair matrix (cf. Fig. S11)**.

To the reviewer's second point, metastability can be used to characterize dynamical repertoires in the brain, operationalized as the standard deviation of the Kuramoto order parameter in the work by Cabral et al. [12] that the reviewer noted. This quantity measures the synchronization across multiple brain regions over time to capture how the brain as a whole transitions between states of synchronization and desynchronization. As it reflects the collective behavior of many regions rather than pairwise interactions, metastability is inherently a higher-order interaction term. While such higher-order interactions fall beyond the already analysis-dense

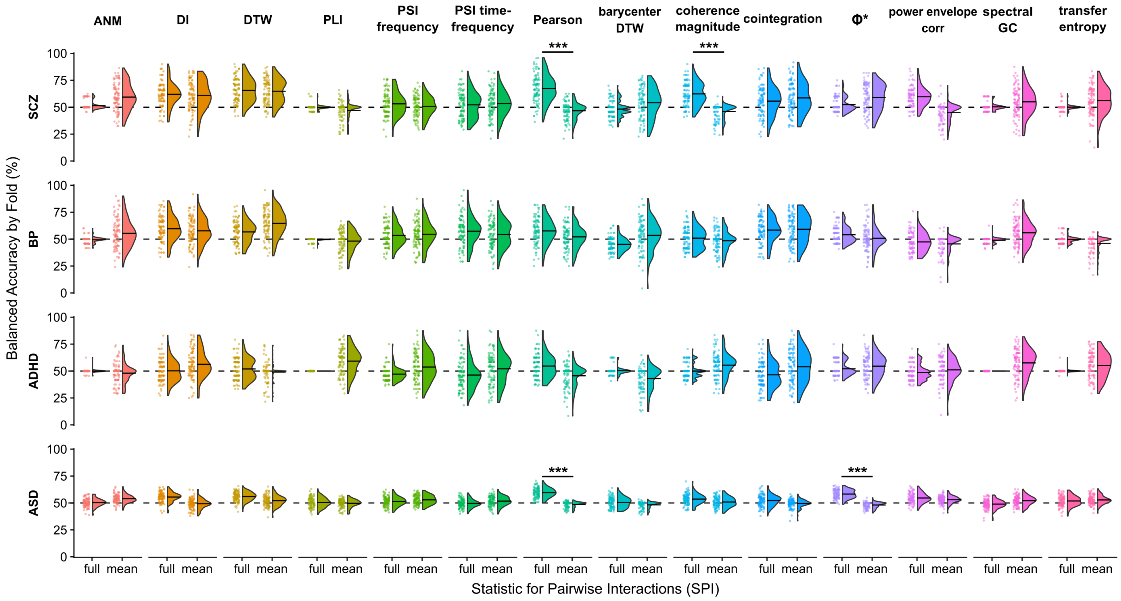

Figure R4: **Comparing classification performance with the full region–region feature space or the brain-wide mean FC for each SPI.** For each SPI, we compared case–control classification performance in each of the four disorders using either the full region–region pair input matrix (left) or the brain-wide average across all region–region pairs (right). Here, we show the distribution of all 100 test folds (10 repeats $\times$ 10 folds) as raincloud plots, where each dot represents one test fold. The solid horizontal line in each half-violin indicates the mean balanced accuracy and the dashed horizontal axis line denotes 50% balanced accuracy. Corrected resampled T-test results are indicated with ***, $P_{\text{corr}} < 0.001$.

scope of this study (as discussed further above in R1.1), we hope that the adaptable systematic framework introduced in our work can be extended to more complex types of dynamical structures, such as this, in future work. We address this point more fully, drawing explicit links to metastability as the reviewer suggests, in the revised Discussion as quoted below.

> Here we focused on five key ways of systematically comparing intra-regional and inter-regional features from an fMRI dataset, but many other representations of spatiotemporal data could also be investigated in future work. This includes quantifying properties of the networks defined by pairwise FC matrices (cf. highly comparative graph analysis [13]), statistics of spatiotemporal patterns (like spirals and traveling waves [14, 15]), and higher-order (beyond pairwise) dependence structures [16]. **For example, statistics such as metastability (based on the standard deviation of the Kuramoto order parameter) could quantify properties of integration and segregation across brain states [12].** More broadly, future work could relax the picture of interacting spatially localized and contiguous brain regions, towards spatially distributed modes, extracted as components through dimensionality reduction [17, 18] or geometrically [19, 20].

1.3. R1 comment #3

R: *Given that the SD and the Pearson correlation matrix have high classification performance, I think it would be interesting to test the performance of the covariance matrix.*

We agree with the reviewer. We computed the covariance from each non-normalized pair of time series and evaluated classification with the same 10-repeat 10-fold cross-validation and linear SVM framework as with all other main analyses; results are shown below, as reproduced from Fig. S12.

Our results indicate that correlation and covariance exhibit similar performance here; there was no statistically significant difference between balanced accuracy obtained using the raw covariance versus normalized Pearson correlation matrices for any of the four disorders (all uncorrected $P > 0.05$ with the corrected resampled T-test [11] as described above). As such, we have opted to focus on the set of 14 SPIs in our original manuscript that all employ z-score normalization before feature computation. In our updated manuscript, we now explicitly note the association between the Pearson correlation and raw covariance matrix (in the revised Results section):

> The significant performance of multiple individual SPIs suggests that inter-regional communication patterns were disrupted in both SCZ and ASD in a way that pairwise FC metrics are generally well suited to capture, consistent with prior work [21, 22]. **Notably, the Pearson correlation coefficient performed well (similarly to the raw covariance matrix, cf. Fig. S12, yielding the highest classification balanced accuracy for SCZ** ($67.3 \pm 12.6\%$, $P_{\text{corr}} = 1 \times 10^{-4}$, **corrected across** 14 **SPIs) and ASD** ($59.5 \pm 4.4\%$, $P_{\text{corr}} = 1 \times 10^{-9}$) **(it was also among the best SPIs for BP, although this was not statistically significant; cf. Fig. S13B[ii]).**

1.4. R1 comment #4

R: *In my opinion, the first two sentences of the introduction are too general and probably not necessary.*

A: We have rephrased the first two sentences in our revised introduction for improved clarity and conciseness:

> Quantifying the dynamical structures in a complex system like the brain can clarify relationships across scales—for example, from individual neuronal populations to macroscopic brain states. In the brain, as with other complex systems, there are myriad ways to represent different types of dynamical patterns, many of which involve discretizing the brain into 'regions' [23, 24].

1.5. R1 comment #5

R: *Lines 260-261: This supports the general use of linear time-series features like the fALFF to parsimoniously summarize relevant properties of rs-fMRI dynamics. I disagree. Figure 2C shows that, for SCZ, the performance of fALFF ranks sixth, behind SD, DFA, periodicity, and mean. Additionally, for ASD, fALFF could not significantly distinguish cases from controls. Or am I missing something?*

A: We thank the reviewer for bringing up this potential source of confusion in our original submission. We intended to highlight that the eleven features that significantly distinguished SCZ and/or ASD cases from controls are generally linear time-series features, with the fALFF just one example along with properties like periodicity and the centroid frequency of the power spectral density ('centroid_freq'). The reviewer is correct that fALFF only significantly distinguished SCZ cases from controls (although we note that, given the variance across test folds for each feature, the differences in mean balanced accuracy among these features should be interpreted with caution). We have revised the sentence accordingly to align more closely with the results presented in Fig. 2B as the reviewer points out:

This supports the general use of linear time-series features—like the centroid frequency of the power spectral density (‘centroid_freq’) or timescale of the autocorrelation function (‘ACF_timescale’)—to parsimoniously summarize relevant properties of rs-fMRI dynamics, while also highlighting the potential utility of the SD to clarify disorder-relevant differences in BOLD signal variance measured across brain regions.

R: *I hope the authors find these comments helpful.*

A: We thank the reviewer for their thoughtful feedback which has strengthened our revised manuscript.

2. Reviewer #2

R: *Thank you for the opportunity to review the manuscript titled Extracting interpretable signatures of whole-brain dynamics through systematic comparison by Bryant and colleagues. The manuscript takes a data-driven approach to systematically compare various intra- and inter-regional dynamics from resting-state fMRI data in the human brain, with a specific focus on their applications in neuropsychiatric disorder classification. Specifically, the authors compare a relatively comprehensive and representative set of time-series and inter-regional connectivity measures (separately and combined) on the regional and whole brain level to benchmark their classification accuracy in 4 psychiatric disorders. They also perform various sensitivity analyses and compare different model selection procedures and classification algorithms. The findings show that linear time-series analysis techniques generally tend to perform well in the classification analysis. Interestingly, the results suggest that although certain measures might outperform others in specific disorders, combined intra- and inter-regional dynamics almost always improve the model performance, especially across 3 out of the 4 included disorders. This demonstrates that the included measures provide complimentary information about disorder-specific brain dynamics. Altogether this manuscript is a novel study that builds on findings from recent studies, where intra- and inter-regional dynamics had been examined separately in the human brain. The study is rigorous and methodologically sound, taking a unique approach to systematically evaluate multiple functional data derivatives and their combinations, assessing their performance in psychiatric disorder classification. The manuscript is well-written and the work is novel, comprehensive, and thorough with interesting findings and compelling sensitivity analysis, providing an important contribution to the literature. I have a couple of minor comments that may help further improve the impact of the study and am happy to recommend a revised version of the manuscript for publication:*

A: We thank the reviewer for their time and positive assessment of our present manuscript, and for their thoughtful suggestions with which to improve the work.

2.1. R2 comment #1

R: *The authors perform a thorough set of sensitivity analyses to ensure that the classification accuracy is independent from covariates and other potential factors including age, sex, and gray matter volume. They also assess whether participants in scanner motion is related to BOLD SD, where they identify a significant association between the two in ASD. Findings of these sensitivity analyses are compelling. However, I am wondering whether the authors have considered examining the potential influence of participants in scanner motion on performance accuracy directly (e.g., training a classifier with age, sex, and mean FD or including mean FD as a covariate in the analysis). It might be worthwhile further examining this given that the mean FD threshold used for data quality control is not necessarily a highly conservative threshold (as the authors acknowledge as well).*

A: We agree that head motion is an important confound in resting-state fMRI analyses. The systematic method we present here is generalizable across scientific domains, though application to the resting-state fMRI space does require careful interpretation of such potential confounding factors that can affect BOLD time series [25]. We have implemented top practices for removing motion confounds from the data using the 'AROMA+2P+GMR' preprocessing method (described in Parkes et al. [26]) to minimize the effect of head motion on the BOLD time series from which we extracted features. However, such preprocessing methods are far from perfect, and as with any BOLD fMRI study, it is impossible to determine the extent to which contributions of head motion have been fully removed from the time series. As the reviewer notes, we reported that BOLD standard deviation (SD) was not correlated with mean framewise displacement (FD) in SCZ (cf. Fig. S5), even though SD was the top-performing feature for SCZ (cf. Fig. 2B) and has previously been linked to head motion [27, 28]—lending credence to the notion that we have identified salient properties of BOLD dynamics that are not driven by head motion.

We agree with the reviewer that it is important to consider the effect of head motion on classification performance directly. We have added these analyses to Fig. S3 in this revised submission to examine sex, age, and/or mean framewise displacement (FD) differences in each case–control comparison, with a focus on the classification performance of models trained on each confound variable with or without the inclusion of BOLD fMRI time-series features. The new figure is reproduced below (cf. Fig. R6) for the reviewer's reference, and we have revised the Methods section to incorporate this new analysis as follows:

> The proportion of males versus females was compared between each disorder and the corresponding control group using a χ^2 test, implemented in R with the `chisq_test` function from *rstatix* (version 0.7.2). We compared age and mean framewise displacement (FD) distributions for each disorder and the corresponding control groups using Wilcoxon rank-sum tests, implemented in R with the `wilcox_test` function from *rstatix* (version 0.7.2). To evaluate how well these confound variables could predict clinical diagnosis on their own, we fit the same 10-repeat 10-fold linear SVM classification pipeline to sex data (with male encoded as 0 and female encoded as 1), age data, and mean FD data. Results from this robustness analysis are depicted in Fig. S3D. We used these classification metrics as a baseline from which we could assess the improvement in case–control distinction using BOLD-derived time-series features. In addition to the performance of these confound variables on their own and that of all the models we evaluated through our main analysis, we additionally evaluated the classification performance of models trained on BOLD time-series features plus confound variables as a robustness analysis. These classification metrics are shown side-by-side in Fig. S3E.

The mean FD was significantly higher in SCZ, BP, and ASD participants relative to their corresponding control participant cohorts, as shown in Fig. S3C (Fig. R6C in this document). When we compared the ability of mean FD to distinguish cases versus controls (as we had done for age and sex in our original submission), we found that head motion alone distinguished cases from controls well for SCZ, and to a lesser extent in BP. This underscores baseline differences in head motion in SCZ in particular, although it is impossible to determine the extent to which head motion artifacts have been removed from any individual resting-state fMRI volume prior to time-series extraction. In light of these new findings inspired by the reviewer's question, we have revised our Results section as follows:

> We also confirmed that the intra-regional dynamics of all significant brain regions distinguished SCZ and BP cases from controls better than a classifier trained solely on age and sex for each disorder (results shown in Table S3), indicating the intra-region dynamical features are capturing disorder-relevant differences beyond these basic demographic features (cf. Fig. S3E; all classification results combining age and sex with features of rs-fMRI dynamics are listed in Table S4). **However, we found that including age, sex, and head motion (quantified as mean framewise displacement, or FD) improved classification performance for some brain regions in SCZ and BP—particularly for SCZ, for which mean FD was significantly higher than controls (cf. Fig. S3C,D). While this does not necessarily indicate that intra-regional dynamics are biased by these confound variables, one should interpret the biological ramifications of region–specific findings with caution given the ability of confound variables to distinguish diagnostic groups.**

As the reviewer asked about the direct effect of head motion on classification performance, we included FD as an input variable (in addition to age and sex) for each model (e.g., left pericalcarine cortex in A_{region}) to compare classification performance with versus without these confound variable terms in Fig. S3E. The classifier trained on age, sex, and FD (labeled 'Age + sex + FD only') tended to outperform models trained solely on time-series features for SCZ, though the union of BOLD time-series features and age, sex, and FD

data yielded top performers in A_{region} and $A_{feature}$ for SCZ. Interestingly, the age+sex+FD model generally outperformed A_{FC} and A_{FC_combo} models in SCZ, which is likely driven in part by the differences in input matrix dimensionality. By contrast, feature-based representations generally yielded superior classification performance (with or without age, sex, and FD) for BP, ADHD, and ASD.

Interpreting the difference in classification performance between features derived from the motion-corrected BOLD time series versus mean FD is difficult without knowing the ground-truth extent to which motion has been cleaned from the time series. This is a caveat common to all BOLD rs-fMRI time-series analysis that warrants consideration for studies applying the generalizable framework described here to such data for the purpose of identifying robust biological insights. Nevertheless, we are encouraged by the finding that BOLD SD was not associated with the mean FD in the SCZ group, which is important for two reasons: first, the BOLD SD was the top-performing feature in $A_{feature}$ for SCZ; and second, BOLD SD has previously been associated with head motion in the scanner [27, 28]. We interpret this to mean that our systematic method can capture properties of resting-state brain dynamics that are not directly associated with confounding factors like head motion.

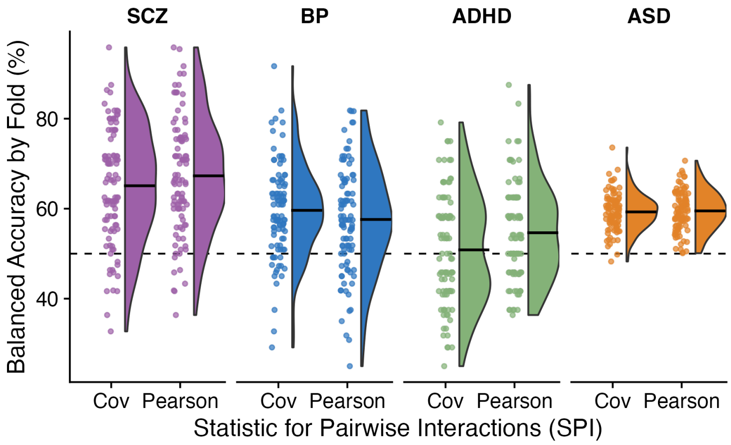

Figure R5: **Raw covariance and normalized Pearson correlation coefficient show comparable case–control classification performance.** For each disorder (SCZ, BP, ADHD, and ASD), we plot the distribution of test balanced accuracy values across 100 folds (10 repeats $\times$ 10 folds) with the same `scikit-learn` pipeline for the raw covariance matrix across all region–pairs ('Cov', left) versus the normalized Pearson correlation matrix ('Pearson', right). The solid horizontal line in each half-violin indicates the mean balanced accuracy and the dashed horizontal axis line denotes 50% balanced accuracy. [Reproduced from Fig. S12.]

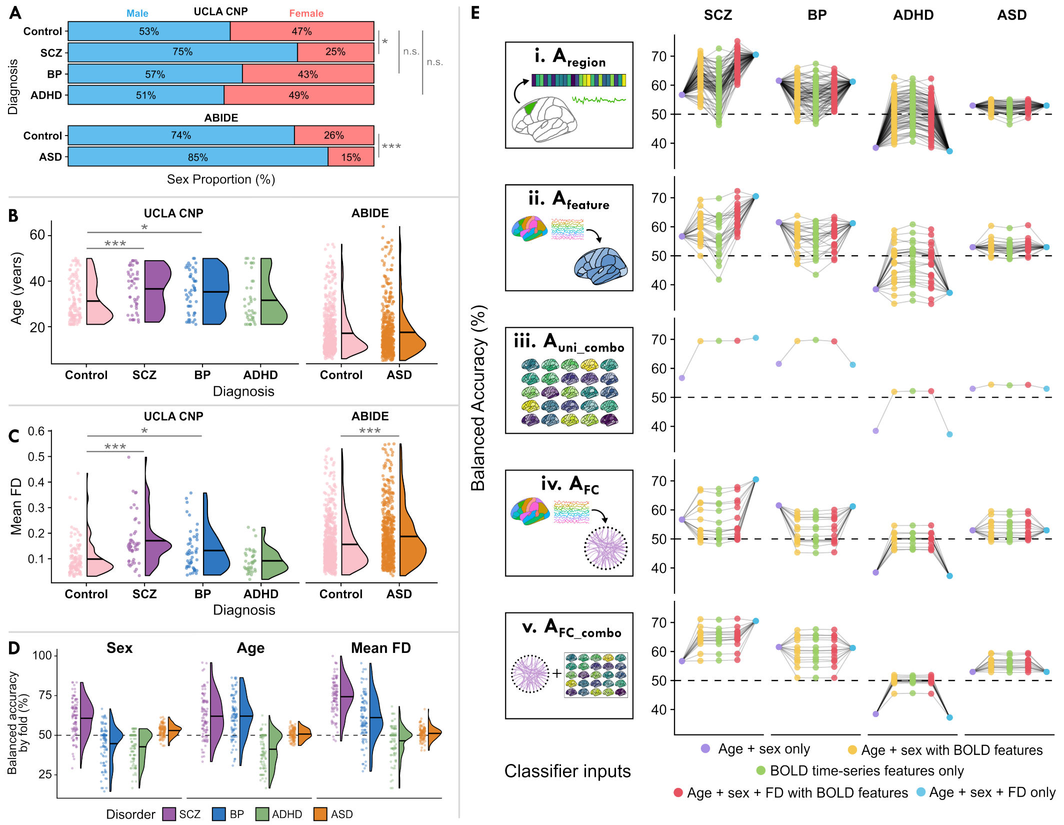

Figure R6: **BOLD fMRI feature-based representations generally improve diagnostic classification beyond age, sex, and mean FD. A.** The percentage of males (blue) and females (red) per diagnostic group is shown for the UCLA CNP cohort (upper) and the ABIDE cohort (lower). Sex proportions were compared for each disorder relative to the corresponding control group using a chi-square test, with significance level indicated as $^{***}P < 0.001$, $^{**}P < 0.01$, $^{*}P < 0.05$, n.s. $P > 0.05$. **B.** The distribution of participant ages is shown as a raincloud plot per diagnostic group for the UCLA CNP cohort (left) and the ABIDE cohort (right). The horizontal line within each half-violin indicates the mean age for the corresponding distribution. Age distributions were compared for each disorder relative to the corresponding control group using a Wilcoxon rank-sum test, with significance level indicated as $^{***}P < 0.001$, $^{**}P < 0.01$, $^{*}P < 0.05$, n.s. $P > 0.05$. **C.** The mean framewise displacement (FD) computed with the method from Power et al. [25] is shown with raincloud plots for all participants in the UCLA CNP (left) and ABIDE cohorts (right). The horizontal line within each half-violin indicates the mean FD for the corresponding group. Mean FD distributions were compared for each disorder relative to the corresponding control group using a Wilcoxon rank-sum test, with significance level indicated as $^{***}P < 0.001$, $^{**}P < 0.01$, $^{*}P < 0.05$. **D.** Case–control classification balanced accuracy is shown for all 100 test folds per each disorder based on participant sex, age, or mean FD. The horizontal line within each half-violin indicates the mean balanced accuracy for the corresponding distribution. The dashed horizontal line in the background denotes 50% balanced accuracy. **E.** For each representation type (rows) and neuropsychiatric disorder (columns), the classification accuracy of each model (e.g., left pericalcarine cortex in A_{region}) is compared using five distinct classier inputs: (1) age + sex only, in purple; (2) age + sex with BOLD time-series features from the given model, in yellow; (3) just BOLD time-series features, in green; (4) age + sex + mean FD with BOLD time-series features, in red; and (5) age + sex + mean FD only. Each dot corresponds to one model (e.g., the left pericalcarine cortex in [i] A_{region}) and lines connect model types to guide visual comparison. The horizontal dashed line in each plot is included to show the chance baseline performance of 50% balanced accuracy.

2.2. R2 comment #2

R: *Classification analysis is applied to each disorder separately. As the authors mention in the manuscript, data harmonization would be required to combine all the data because of differences in sample size and acquisition sites. However, I am wondering whether it would be possible to combine the UCLA sample for the data acquired in one of the two acquisition sites and assess the model performance, especially for the* A_{FC_combo} *analysis. This might be particularly interesting as it would test the classification accuracy of the included measures in data with multiple categories (rather than binary labels).*

A: We thank the reviewer for addressing the prospect of comparing transdiagnostic versus disorder-specific properties of resting-state dynamical structure. Our focus for the present work is on demonstrating the systematic methodological approach to extracting dynamical structure of different types, using relatively simple linear classification pipelines—with detailed optimizations for disorder classification left to the scope of future work. While transdiagnostic analyses across multiple disorders (and/or disorder subtypes) lies beyond the scope of the present study, we agree that this presents an interesting avenue for future work. We now address this explicitly in the Discussion:

> While this work presents a systematic methodological framework that is flexible and generalizable across domains, future studies aiming to identify disorder-specific biomarkers will need to evaluate their findings in external validation datasets to thoroughly assess the validity of a given brain region or time-series feature. **Moreover, future work could extend beyond the binary case–control classification paradigm to examine transdiagnostic versus disorder-specific properties of resting-state dynamical structure [29, 30].**

As a preliminary inroad in that direction, we performed new multi-class analyses using pairwise coupling statistics (in representations $\mathrm{A_{FC}}$ and $\mathrm{A_{FC\_combo}}$) with all four groups in the UCLA CNP dataset: (1) control, (2) SCZ, (3) BP, and (4) ADHD, which we now include as a supplementary figure (Fig. S13) that has been reproduced below in Fig. R7.

These results are consistent with the analyses presented in Fig. 4A of the main text—namely, that metrics like the Pearson correlation coefficient, dynamic time warping (DTW), directed information (DI), Φ^*, and the coherence magnitude are the top-performing properties of functional connectivity, with or without the inclusion of whole-brain local dynamics. While we wish to reiterate that this forms a preliminary analysis on a topic that warrants its own paper entirely, we briefly address these findings in the Results section now (relevant sentence bolded):

> In order to better understand the types of SPIs that performed well for SCZ and ASD, we computed an empirical SPI similarity index ($|\rho_{SPI}|$) using the same method as implemented in $\mathrm{A_{feature}}$ above (i.e., computed across all controls as well as SCZ and ASD participants; cf. Sec. 3.2). As shown in Fig. 3B (and listed in Table S13), the Pearson correlation coefficient behaved most similarly to dynamic time warping (DTW, similarity index $|\rho_{SPI}| = 0.40$), which incorporates shifts and dilations between a pair of time series [31, 32] and has previously been suggested as a promising alternative to standard correlation for rs-fMRI analysis [33]. Other top-performing SPIs included directed information ('DI', [34]) and Φ^* [35], which captured different types of pairwise coupling compared to the Pearson correlation coefficient and DTW, as shown in Fig. 3B, and warrant further investigation in future work. **We found similar results when assessing multi-diagnosis classification performance in the UCLA CNP cohort (cf. Fig. S13)**. Ultimately, the Pearson correlation coefficient was both the top-performing SPI based on case–control classification and the most parsimonious, underscoring the utility of measuring linear, synchronous inter-regional coupling under a Gaussian assumption to

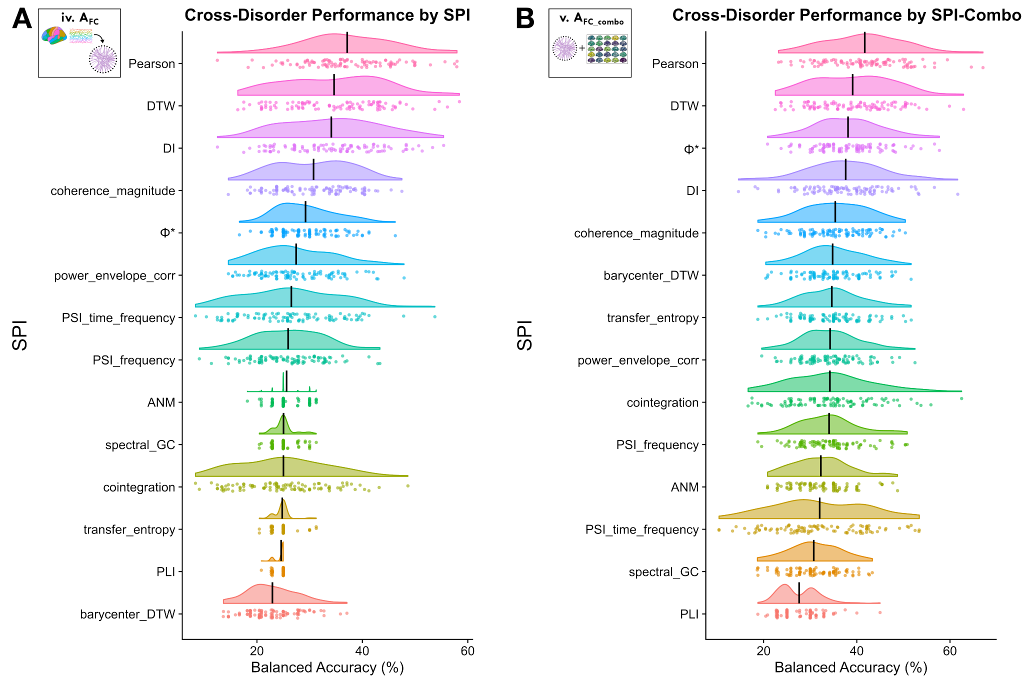

Figure R7: **Cross-disorder classification analysis in the UCLA CNP cohort based on pairwise functional connectivity.** The out-of-sample balanced accuracy distribution (averaged across SCZ, BP, ADHD, and Control participants in the UCLA CNP cohort) is shown for each SPI on its own (**A**, A_{FC}) or with the inclusion of whole-brain local dynamics (**B**, A_{FC_combo}). Each dot corresponds to one test fold (for a total of 100 data points, across 10 repeats $\times$ 10 folds), and the vertical black line in each half-violin corresponds to the mean balanced accuracy across test folds.

capture disorder-relevant pairwise dependence structures from rs-fMRI.

3. Reviewer #3

R: *The paper is well-written and asks an interesting question on the predictive value of fMRI dynamics for classifying disease vs. control. Three types of disease are used, and wide variety of models are tested to answer the question comprehensively. Here are my main comments:*

3.1. R3 comment #1

R: *Here are my main comments: 1. significance: it was not clear what is the state-of-the-art predictive performance for these disease classification problems, which features (univariate or FC) achieve that, and whether dynamic features are helping improve over that. Figure 3 shows Pearson FC has highest accuracy among the dynamic SPI measures.*

A: The main goal of our manuscript is to develop a flexible and interpretable classification framework for multivariate dynamics that enables systematic comparison across different types of quantitative dynamical representations. Maximizing classification performance was not a core aim of this work, but we acknowledge that comparison to prior work can help to contextualize our results. Within the last five years, some studies have reported >90% case–control classification accuracy in each of the four neuropsychiatric disorders considered in the present study, typically combining deep neural network approaches with smaller sample sizes [36–40]. However, small sample sizes and complex classifiers can lead to overestimated predictive performance in neuropsychiatric disorders [41], which is problematic in this scientific setting in which understanding and generalizability are paramount [42].

Indeed, two recent studies rigorously evaluated multiple different types of classifiers for hundreds [43] or thousands [44] of participants with major depressive disorder or healthy controls, finding in both cases that the maximum classification accuracy failed to surpass 65%. Another group working with the same ASD dataset as examined in this manuscript (from the ABIDE I [45] and II [46] cohorts) reported 62% accuracy, with similar values for other large ASD datasets [47]. Moreover, recent work with a large multi-sample SCZ cohort found a maximum of 77.3% case–control classification accuracy [48]. As such, the classification performance in this study (with simple linear classifiers and minimal parameter tuning) is comparable to that of methodologically rigorous large-scale studies. We therefore leave performance optimization as an opportunity for future work, keeping interpretability as the focus. We have improved our description of this goal in our updated Introduction, as quoted below:

> Despite the improvements to in-sample classification performance often afforded by this increased complexity, core goals of neuroimaging-based classification studies include: (1) unpacking the neuroanatomical and functional underpinnings of a given disorder; and (2) developing a generalizable classification framework that aids the diagnosis of future (unseen) patients [49–53]. Here, we implement linear support vector machine (SVM) classifiers to combine features based on clear algorithms derived from interdisciplinary literature, aiming to compare and contrast the types of underlying dynamical processes that distinguish brain activity across disorders—rather than absolute classification performance [54, 55]. Broadly, insights into the dynamical processes that are altered can deepen understanding of mechanisms underlying disease pathogenesis and progression, potentially yielding clinically translatable biomarkers [56, 57].

And with further additions to our revised Discussion:

> The goal of this study was to compare within and across interpretable representations of rs-fMRI dynamics, with a focus on results obtained with the linear SVM classifier for simplicity. We explored

alternative classifiers that implement SVM with an RBF kernel or L1 ('LASSO') regularization, random forest, or gradient boosting (cf. Fig. S7) and did not observe general performance improvements, although it is possible that combining other nonlinear classifiers with hyperparameter optimization could more sensitively discriminate among features in a given disorder if nonlinear boundaries are present. Maximizing case–control classification performance was not an explicit aim of this study, although we note that our findings sit within the range of recent rigorous large-scale classification studies [43, 44, 47]. Future work could expand upon this generalizable framework to optimize performance in a given application setting.

R: *It is also not clear if any scientific insight can be provided based on feature importances of the fitted models.*

A: Note that none of our analyses computed feature importance scores derived from fitted multivariate models. Indeed, the systematic method we introduce presents a conceptual departure from concatenating all features into a single classifier from which feature weights are extracted for downstream interpretation. This was a deliberate choice, as interpreting such feature importance scores can be problematic in large models containing multicollinearity among input features [58]. Our modular approach incorporates incremental complexity from individual regions up to integrated whole-brain maps of local dynamics and pairwise coupling in a way that clarifies the types of structures that are informative in a given application as a whole—without probing the specific parameters from which any individual model was constructed. We now clarify this point in the Discussion:

> Future applications of this systematic framework might also consider more nuanced data fusion techniques beyond simple matrix concatenation to properly combine heterogeneous input data types (e.g., multiple brain regions; local dynamics versus pairwise coupling) into one classifier [59, 60]. Our modular approach incorporates incremental complexity from individual brain regions up to integrated whole-brain maps of local dynamics and pairwise coupling, such that results are interpreted based on the overall performance of a given model. While we did not explore feature importance scores due to intrinsic issues with multicollinearity among input features, future work could incorporate dimensionality reduction techniques to mitigate this collinearity and specifically interpret individual feature weights (e.g., linear SVM coefficients) to compare relative contributions of different brain regions and/or temporal signatures to a given decision boundary.

3.2. R3 comment #2

R: *Approach: I have serious concerns about potential overfitting in the results. The sample sizes are modest, the feature set is huge, and the authors are using linear SVM with the same default regularization (see lines 807-815 in page 27). Since the amount of regularization is not tuned to model size, it is possible that larger models with many features will be favored, which matches the empirical results.*

A: We thank the reviewer for raising the point of overfitting, which is crucial to address comprehensively in any statistical learning problem. First, we reiterate that all of our reported results are derived from completely unseen data, as we measure balanced accuracy across test folds in a repeated cross-validation procedure. This ensures that the higher-performing models that we reported are the result of optimistic overfitting, as such overfitting would yield poor generalizability and therefore lower out-of-sample performance.

Model overfitting is a particular concern in human neuroimaging studies, which often have $p \gg n$ (i.e., far more features than samples) [61]. Indeed, models with greater degrees of freedom (i.e., more input features) are more prone to overfitting, as per the 'curse of dimensionality' [62]. Our empirical findings indicate

that classification performance does *not* increase monotonically with model size (cf. Fig R8 below). For example, in the SCZ group, we found in Fig. 2E that one brain region (feature space of 25 dimensions) and one time-series feature (feature space of 82 dimensions) outperformed the classifier with all brain regions and all time-series features (A_{uni_combo}, feature space of 2050 dimensions). Similar findings were observed for ASD (and ADHD, although none of the balanced accuracy metrics were statistically significant). By contrast, the A_{uni_combo} classifier showed the maximal performance for BP, suggesting that combining brain regions and time-series features into one classifier provided additional signal that generalized beyond the training set.

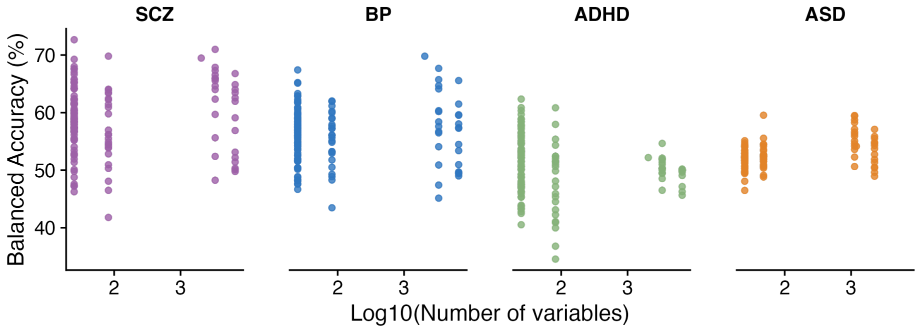

Figure R8: **Classification performance does not increase monotonically with the number of features associated with each representation type.** For each disorder, the mean cross-validated balanced accuracy is plotted versus the $\log_{10}$-transformed number of input features to the linear SVM classifier per representation type. For example, the A_{region} representation includes models with a total of 25 features inputted to the classifier—corresponding to the 25 univariate time-series features measured per region.

We now more clearly highlight this example of how model size and classification performance vary in the revised Discussion:

> Although the BOLD SD was contained within the combined region × feature matrix evaluated in the combination classifier A_{uni_combo}, increasing the number of input attributes to a classifier does not necessarily improve its performance due to overfitting beyond the 'latent dimensionality' of the dataset, defined as the number of meaningful variables inferred from the data that capture underlying essential patterns [63, 64].

We also expand upon this point in our revised Results, along with our steps to account for potential overfitting and assess generalizability with cross-validation:

> In contrast to the previous two reduced representations of intra-regional dynamics across the brain—in either a single region (A_{region}) or of a single time-series property ($A_{feature}$)—we next asked if a more comprehensive representation that integrates multiple time-series properties of resting-state activity measured from all brain regions could improve case–control classification performance. This entails a much higher-dimensional representation of brain-wide dynamics (2050 dimensions for the UCLA CNP cohort: SCZ, BP, and ADHD, and 1200 dimensions for the ABIDE ASD cohort). Higher-dimensional input spaces are more prone to overfitting due to greater complexity from more free

parameters (described as the 'curse of dimensionality' [62]), and potentially from noise accumulation in the setting of noisy features [65]. To account for potential overfitting, we applied 10-repeat 10-fold cross-validation and report out-of-sample performance to evaluate the generalizability of a model's predictive performance [66]. An improvement in performance (assessed via balanced accuracy) in this representation would therefore suggest that multiple properties of intra-regional activity measured across multiple spatial locations are important for characterizing a given disorder, in a way that cannot be reduced to a single region or single time-series feature.

R: *The discussion on variable selection is diffuse, and I would like to see the robustness of the findings when a state-of-the-art regularization ML method (LASSO, Ridge, even random forests or gradient boosting which are robust to default tuning parameter choices) is used.*

A: We agree that comparing different machine learning methods provides important insights into the types of properties that distinguish groups in a given analysis—for example, whether a linear or nonlinear decision boundary better captures case–control differences. We wish to clarify that we included a robustness analysis for comparing amongst different (1) classifier types and (2) tuning parameters, using a nested cross-validation approach as summarized in Fig. S7A. Our empirical findings support the use of the linear kernel for our SVM classifier with a constant regularization parameter of $C = 1$, though future work could further explore performance across models with different regularization parameters evaluated with different hyperparameter tuning methods.

We have improved the corresponding section of the Methods in our revised submission to more clearly describe this robustness analysis and our interpretations:

As a robustness analysis, we subsequently compared the performance of each model (e.g., the left pericalcarine cortex in A_{region}) with the same 10-repeat 10-fold cross-validation as in the main analysis using each of: a linear SVM with L1 ('LASSO') coefficient regularization [67] and squared-hinge loss, an SVM with a radial basis kernel, a random forest ensemble, or a gradient boosted decision tree ensemble. The standard linear SVM with hinge loss and without L1 coefficient regularization yielded the highest classification performance overall (cf. Fig. S7A), and as this linear SVM represented the most parsimonious and interpretable of the evaluated models, we focus on classification metrics from this model throughout the paper. We also evaluated the effect of nested cross-validation to tune the regularization parameter, C, across values ranging between [0.0001, 100]—as well as the inclusion (or exclusion) of inverse probability weighting—finding that this also did not generally improve performance beyond our *a priori* parameter settings of $C = 1$ and applying inverse probability weighting (cf. Fig. S7B and Table S17).

3.3. R3 comment #3

R: *Validation: Since there is no tuning parameter in the model, I could not understand how cross-validation (CV) is being used. In machine learning literature, it is customarty to reporst test or validation set errors, rather than CV-errors. It would be good to report out-of-sample performances on data that never touched by the model.*

A: We thank the reviewer for highlighting the importance of reporting out-of-sample classification performance to assess model generalizability. We wish to clarify that we use the term 'cross-validation' in the present study to indicate repeatedly partitioning the data into test folds [68], such that for each iteration, we train the model on the training subset and evaluate the model on the held-out test subset (comprised of the remaining unseen participants), with all classification results reported from the unseen test subsets. Therefore, all

results reported in this study were derived from models trained on training portions of the data and tested on unseen data to which the model never had access. This statistically hygienic practice ensures that our results cannot be enhanced by potential overfitting in the training portions. We have clarified this point as follows in the Methods of our revised manuscript as follows:

> Classifiers were evaluated using stratified 10-fold cross-validation (CV) such that the hyperplane was defined based on the training data and used to predict the diagnostic labels of the unseen test data for each fold. In order to reduce outlier-driven skews in time-series feature distributions, each training fold was normalized using the scaled outlier-robust sigmoidal transformation described in [69] and the same normalization was applied to each test fold to prevent data leakage [70]. We confirmed that this reduced more distributional skew of many univariate and pairwise time-series features compared to z-score normalization, as shown in Fig. S15. To ensure that results were not driven by any one specific CV split, each 10-fold CV procedure was repeated 10 times [71–73], yielding 100 out-of-sample balanced accuracy metrics per model.

R: *Finally, balanced accuracy measures depend on choice of decision threshold. So reporting area under ROC or precision-recall curve is perhaps a more fair metric when comparing models.*

We agree that metrics like area under the curve (AUC), which aggregate over a range of decision thresholds, can have advantages over metrics like balanced accuracy derived from a single learned threshold. In our analysis, we compared the mean out-of-sample balanced accuracy versus mean AUC for each evaluated model that yielded balanced accuracy of 50% or greater, finding that the Pearson correlation ranged from $R = 0.77$ (ADHD) to $R = 0.97$ (ASD). The high similarity between these two metrics is visually depicted below (cf. Fig R9) for the reviewer's reference.

Given the reduced computational demand and clearer interpretations for a binary classifier like a linear SVM with balanced accuracy versus the AUC [74], we favored reporting the simpler-to-interpret balanced accuracy here. We now summarize this point more clearly in our revised Methods:

> While the balanced accuracy is only computed for the default decision threshold for each classifier, we also computed the area under the receiving operator characteristic curve (AUC) for each test fold to evaluate model performance across thresholds. Models achieving greater than 50% balanced accuracy (i.e., better than chance) showed similar performance between balanced accuracy and AUC (Pearson's R between $0.77 - 0.97$), so we focus on balanced accuracy in this work to align with the binary predictions of diagnostic group [75].

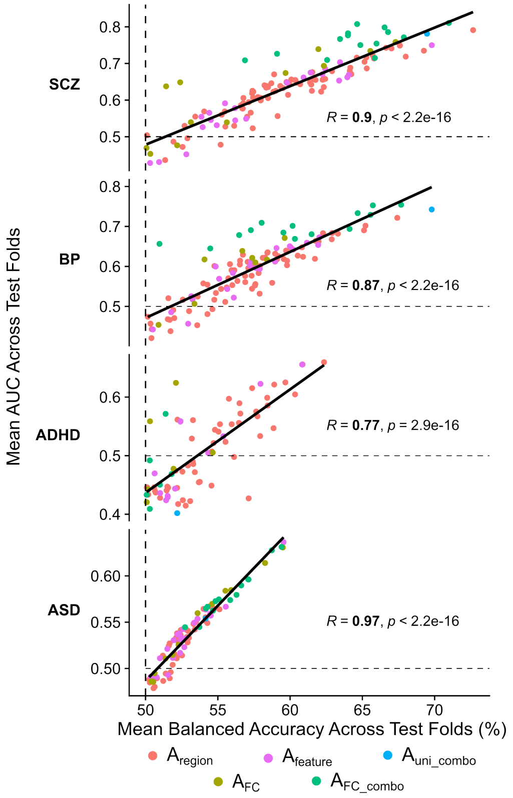

Figure R9: **Balanced accuracy and the area under the curve (AUC) yield comparable results across representations.** For each of the four neuropsychiatric disorders, the mean out-of-sample AUC is plotted against the mean balanced accuracy for each model. Points represent a single model and are colored by representation type (e.g., one orange point corresponds to the left entorhinal cortex for A_{region}). Values were truncated at 50% balanced accuracy or greater to focus on models that performed better than chance.

References

[1] A. Ponce-Alvarez, L. Uhrig, N. Deco, et al. Macroscopic quantities of collective brain activity during wakefulness and anesthesia. *Cerebral cortex* **32**, 298 (2022).

[2] K. M. Aquino, B. D. Fulcher, L. Parkes, K. Sabaroedin, and A. Fornito. Identifying and removing widespread signal deflections from fMRI data: Rethinking the global signal regression problem. *Neuroimage* **212**, 116614 (2020).

[3] S. Tanabe, A. Bo, M. White, et al. Cohort study of electroencephalography markers of amyloid-tau-neurodegeneration pathology. *Brain Communications* **2**, fcaa099 (2020). Number: 2.

[4] D. Lei, W. H. Pinaya, J. Young, et al. Integrating machining learning and multimodal neuroimaging to detect schizophrenia at the level of the individual. *Human brain mapping* **41**, 1119 (2020).

[5] Z. Dai, C. Yan, Z. Wang, et al. Discriminative analysis of early Alzheimer's disease using multi-modal imaging and multi-level characterization with multi-classifier (M3). *Neuroimage* **59**, 2187 (2012).

[6] X. Ding, Y. Yang, E. A. Stein, and T. J. Ross. Combining multiple resting-state fMRI features during classification: Optimized frameworks and their application to nicotine addiction. *Frontiers in human neuroscience* **11**, 362 (2017).

[7] L. Xia, A. S. Malik, and A. R. Subhani. A physiological signal-based method for early mental-stress detection. In *Cyber-Enabled Intelligence*, pp. 259–289. Taylor & Francis (2019).

[8] B. Goertzel, J. Pitt, M. Ikle, C. Pennachin, and L. Rui. Glocal memory: A critical design principle for artificial brains and minds. *Neurocomputing* **74**, 84 (2010).

[9] O. M. Cliff, A. G. Bryant, J. T. Lizier, N. Tsuchiya, and B. D. Fulcher. Unifying pairwise interactions in complex dynamics. *Nature Computational Science* pp. 1–11 (2023).

[10] Z. S. Saad, R. C. Reynolds, H. J. Jo, et al. Correcting brain-wide correlation differences in resting-state fmri. *Brain connectivity* **3**, 339 (2013).

[11] T. Henderson. hendersontrent/correctr: v0.1.2 (2022). URL: https://doi.org/10.5281/zenodo.7450146.

[12] J. Cabral, E. Hugues, O. Sporns, and G. Deco. Role of local network oscillations in resting-state functional connectivity. *Neuroimage* **57**, 130 (2011).

[13] R. L. Peach, A. Arnaudon, J. A. Schmidt, et al. HCGA: Highly comparative graph analysis for network phenotyping. *Patterns* **2**, 100227 (2021). Number: 4.

[14] Y. Xu, X. Long, J. Feng, and P. Gong. Interacting spiral wave patterns underlie complex brain dynamics and are related to cognitive processing. *Nature human behaviour* pp. 1–20 (2023).

[15] R. G. Townsend and P. Gong. Detection and analysis of spatiotemporal patterns in brain activity. *PLoS computational biology* **14**, e1006643 (2018).

[16] A. Santoro, F. Battiston, M. Lucas, G. Petri, and E. Amico. Higher-order connectomics of human brain function reveals local topological signatures of task decoding, individual identification, and behavior. *bioRxiv* pp. 2023–12 (2023).

[17] N. W. Bailey, B. D. Fulcher, B. Caldwell, et al. Uncovering a stability signature of brain dynamics

associated with meditation experience using massive time-series feature extraction. *Neural networks* (2023).

[18] A. H. Williams, T. H. Kim, F. Wang, et al. Unsupervised discovery of demixed, low-dimensional neural dynamics across multiple timescales through tensor component analysis. *Neuron* **98**, 1099 (2018).

[19] E. J. Müller, B. R. Munn, K. M. Aquino, J. M. Shine, and P. A. Robinson. The music of the hemispheres: Cortical eigenmodes as a physical basis for large-scale brain activity and connectivity patterns. *Frontiers in human neuroscience* **16**, 1062487 (2022).

[20] J. C. Pang, K. M. Aquino, M. Oldehinkel, et al. Geometric constraints on human brain function. *Nature* pp. 1–9 (2023).

[21] M. Greicius. Resting-state functional connectivity in neuropsychiatric disorders. *Current opinion in neurology* **21**, 424 (2008).

[22] T. Takamura and T. Hanakawa. Clinical utility of resting-state functional connectivity magnetic resonance imaging for mood and cognitive disorders. *Journal of neural transmission* **124**, 821 (2017).

[23] L. Pessoa. Understanding brain networks and brain organization. *Physics of life reviews* **11**, 400 (2014).

[24] C. Horien, A. S. Greene, R. T. Constable, and D. Scheinost. Regions and connections: Complementary approaches to characterize brain organization and function. *The Neuroscientist* **26**, 117 (2020).

[25] J. D. Power, K. A. Barnes, A. Z. Snyder, B. L. Schlaggar, and S. E. Petersen. Spurious but systematic correlations in functional connectivity MRI networks arise from subject motion. *Neuroimage* **59**, 2142 (2012).

[26] L. Parkes, B. Fulcher, M. Yücel, and A. Fornito. An evaluation of the efficacy, reliability, and sensitivity of motion correction strategies for resting-state functional mri. *Neuroimage* **171**, 415 (2018).

[27] J. D. Power, M. Plitt, T. O. Laumann, and A. Martin. Sources and implications of whole-brain fMRI signals in humans. *Neuroimage* **146**, 609 (2017).

[28] J. Sheng, L. Zhang, J. Feng, et al. The coupling of BOLD signal variability and degree centrality underlies cognitive functions and psychiatric diseases. *Neuroimage* **237**, 118187 (2021).

[29] F. Brandl, B. Weise, S. Mulej Bratec, et al. Common and specific large-scale brain changes in major depressive disorder, anxiety disorders, and chronic pain: a transdiagnostic multimodal meta-analysis of structural and functional mri studies. *Neuropsychopharmacology* **47**, 1071 (2022).

[30] Y. Liu, M. S. Mellem, H. Gonzalez, et al. Highly predictive transdiagnostic features shared across schizophrenia, bipolar disorder, and adhd identified using a machine learning based approach. *bioRxiv* p. 453951 (2018).

[31] H. Sakoe and S. Chiba. Dynamic programming algorithm optimization for spoken word recognition. *IEEE transactions on signal processing* **26**, 43 (1978).

[32] F. Itakura. Minimum prediction residual principle applied to speech recognition. *IEEE Transactions on acoustics, speech, and signal processing* **23**, 67 (1975).

[33] A. C. Linke, L. E. Mash, C. H. Fong, et al. Dynamic time warping outperforms Pearson correlation in detecting atypical functional connectivity in autism spectrum disorders. *Neuroimage* **223**, 117383

(2020).

[34] J. Massey. Causality, feedback and directed information. In *Proceedings of the International Symposium on Information Theory and Its Applications (ISITA-90)*, pp. 303–305 (1990).

[35] M. Oizumi, S.-i. Amari, T. Yanagawa, N. Fujii, and N. Tsuchiya. Measuring integrated information from the decoding perspective. *PLoS computational biology* **12**, e1004654 (2016).

[36] M. Ghanbari, A. H. Pilevar, and N. Bathaeian. Diagnosis of schizophrenia using brain resting-state fmri with activity maps based on deep learning. *Signal, Image and Video Processing* **17**, 267 (2023).

[37] X. Chen, J. Zhou, P. Ke, et al. Classification of schizophrenia patients using a graph convolutional network: A combined functional mri and connectomics analysis. *Biomedical Signal Processing and Control* **80**, 104293 (2023).

[38] V. Khullar, K. Salgotra, H. P. Singh, and D. P. Sharma. Deep learning-based binary classification of adhd using resting state mr images. *Augmented Human Research* **6**, 5 (2021).

[39] F. Z. Subah, K. Deb, P. K. Dhar, and T. Koshiba. A deep learning approach to predict autism spectrum disorder using multisite resting-state fmri. *Applied Sciences* **11**, 3636 (2021).

[40] F. Ahmad, I. Ahmad, and Y. Guerrero-Sánchez. Classification of schizophrenia-associated brain regions in resting-state fmri. *The European Physical Journal Plus* **138**, 1 (2023).

[41] C. Flint, M. Cearns, N. Opel, et al. Systematic misestimation of machine learning performance in neuroimaging studies of depression. *Neuropsychopharmacology* **46**, 1510 (2021).

[42] B. W. Brunton and M. Beyeler. Data-driven models in human neuroscience and neuroengineering. *Current opinion in neurobiology* **58**, 21 (2019).

[43] N. R. Winter, J. Blanke, R. Leenings, et al. A systematic evaluation of machine learning–based biomarkers for major depressive disorder. *JAMA psychiatry* **81**, 386 (2024).

[44] V. Belov, T. Erwin-Grabner, M. Aghajani, et al. Multi-site benchmark classification of major depressive disorder using machine learning on cortical and subcortical measures. *Scientific reports* **14**, 1084 (2024).

[45] A. Di Martino, C.-G. Yan, Q. Li, et al. The autism brain imaging data exchange: Towards a large-scale evaluation of the intrinsic brain architecture in autism. *Molecular psychiatry* **19**, 659 (2014).

[46] A. Di Martino, D. O'Connor, B. Chen, et al. Enhancing studies of the connectome in autism using the Autism Brain Imaging Data Exchange II. *Scientific data* **4**, 170010 (2017).

[47] T. Itahashi, A. Yamashita, Y. Takahara, et al. Generalizable and transportable resting-state neural signatures characterized by functional networks, neurotransmitters, and clinical symptoms in autism. *Molecular Psychiatry* pp. 1–13 (2024).

[48] T. Kawashima, A. Yamashita, Y. Yoshihara, et al. Generalisable functional imaging classifiers of schizophrenia have multifunctionality as trait, state, and staging biomarkers. *medRxiv* pp. 2024–01 (2024).

[49] K. H. Taber, R. A. Hurley, and S. C. Yudofsky. Diagnosis and treatment of neuropsychiatric disorders. *Annual review of medicine* **61**, 121 (2010).

[50] M. S. Breen, D. J. Stein, and D. S. Baldwin. Systematic review of blood transcriptome profiling in neuropsychiatric disorders: Guidelines for biomarker discovery. *Human psychopharmacology: Clinical and experimental* **31**, 373 (2016).

[51] W. Yassin, H. Nakatani, Y. Zhu, et al. Machine-learning classification using neuroimaging data in schizophrenia, autism, ultra-high risk and first-episode psychosis. *Translational psychiatry* **10**, 278 (2020).

[52] N. Khalili-Mahani, S. A. Rombouts, M. J. van Osch, et al. Biomarkers, designs, and interpretations of resting-state fMRI in translational pharmacological research: A review of state-of-the-art, challenges, and opportunities for studying brain chemistry. *Human brain mapping* **38**, 2276 (2017).

[53] C.-W. Woo, L. J. Chang, M. A. Lindquist, and T. D. Wager. Building better biomarkers: Brain models in translational neuroimaging. *Nature neuroscience* **20**, 365 (2017).

[54] M. Markicevic, B. D. Fulcher, C. Lewis, et al. Cortical excitation:inhibition imbalance causes abnormal brain network dynamics as observed in neurodevelopmental disorders. *Cerebral cortex* **30**, 4922 (2020).

[55] Y. Chen. Altered functional dynamics gradient in schizophrenia with cigarette smoking. *Cerebral cortex* **33**, 7185 (2023).

[56] K. Specht. Current challenges in translational and clinical fmri and future directions. *Frontiers in psychiatry* **10**, 924 (2020).

[57] P. J. Uhlhaas and W. Singer. Neuronal dynamics and neuropsychiatric disorders: Toward a translational paradigm for dysfunctional large-scale networks. *Neuron* **75**, 963 (2012).

[58] L. Toloşi and T. Lengauer. Classification with correlated features: unreliability of feature ranking and solutions. *Bioinformatics* **27**, 1986 (2011).

[59] X. Zhu, H. Li, H. T. Shen, et al. Fusing functional connectivity with network nodal information for sparse network pattern learning of functional brain networks. *Information fusion* **75**, 131 (2021).

[60] J. Y. Hansen, G. Shafiei, K. Voigt, et al. Integrating multimodal and multiscale connectivity blueprints of the human cerebral cortex in health and disease. *PLoS biology* **21**, e3002314 (2023).

[61] T. Hastie and R. Tibshirani. Efficient quadratic regularization for expression arrays. *Biostatistics* **5**, 329 (2004).

[62] M. Verleysen and D. François. The curse of dimensionality in data mining and time series prediction. In *Computational Intelligence and Bioinspired Systems: 8th International Work-Conference on Artificial Neural Networks, IWANN 2005, Vilanova i la Geltrú, Barcelona, Spain, June 8-10, 2005. Proceedings 8*, pp. 758–770. Springer (2005).

[63] G. Hughes. On the mean accuracy of statistical pattern recognizers. *IEEE transactions on information theory* **14**, 55 (1968).

[64] G. V. Trunk. A problem of dimensionality: A simple example. *IEEE transactions on pattern analysis and machine intelligence* pp. 306–307 (1979).

[65] H. Jamalabadi, S. Alizadeh, M. Schönauer, C. Leibold, and S. Gais. Classification based hypothesis

testing in neuroscience: Below-chance level classification rates and overlooked statistical properties of linear parametric classifiers. *Human brain mapping* **37**, 1842 (2016).

[66] D. Berrar. Cross-validation. In S. Ranganathan, M. Gribskov, K. Nakai, and C. Schönbach, eds., *Encyclopedia of Bioinformatics and Computational Biology*, pp. 542–545. Academic Press, Oxford (2019).

[67] R. Tibshirani, M. Saunders, S. Rosset, J. Zhu, and K. Knight. Sparsity and smoothness via the fused lasso. *Journal of the Royal Statistical Society Series B: Statistical Methodology* **67**, 91 (2005).

[68] B. Ghojogh and M. Crowley. The theory behind overfitting, cross validation, regularization, bagging, and boosting: tutorial. *arXiv preprint arXiv:1905.12787* (2019).

[69] B. D. Fulcher and N. S. Jones. hctsa: Computational framework for automated time-series phenotyping using massive feature extraction. *Cell systems* **5**, 527 (2017). Number: 5.

[70] N. Bussola, A. Marcolini, V. Maggio, G. Jurman, and C. Furlanello. AI slipping on tiles: Data leakage in digital pathology. In *Pattern Recognition. ICPR International Workshops and Challenges: Virtual Event, January 10–15, 2021, Proceedings, Part I*, pp. 167–182. Springer (2021).

[71] G. Varoquaux, P. R. Raamana, D. A. Engemann, et al. Assessing and tuning brain decoders: Cross-validation, caveats, and guidelines. *Neuroimage* **145**, 166 (2017).

[72] J. Li, R. Kong, R. Liégeois, et al. Global signal regression strengthens association between resting-state functional connectivity and behavior. *Neuroimage* **196**, 126 (2019).

[73] B. Larsen, Z. Cui, A. Adebimpe, et al. A developmental reduction of the excitation:inhibition ratio in association cortex during adolescence. *Science advances* **8** (2022).

[74] J. Muschelli III. Roc and auc with a binary predictor: a potentially misleading metric. *Journal of classification* **37**, 696 (2020).

[75] P. Thölke, Y.-J. Mantilla-Ramos, H. Abdelhedi, et al. Class imbalance should not throw you off balance: Choosing the right classifiers and performance metrics for brain decoding with imbalanced data. *Neuroimage* **277**, 120253 (2023).
